# Identification and functional analysis of olfactory receptor family reveal unusual characteristics of the olfactory system in the migratory locust

**DOI:** 10.1007/s00018-015-2009-9

**Published:** 2015-08-12

**Authors:** Zhifeng Wang, Pengcheng Yang, Dafeng Chen, Feng Jiang, Yan Li, Xianhui Wang, Le Kang

**Affiliations:** State Key Laboratory of Integrated Management of Pest Insects and Rodents, Institute of Zoology, Chinese Academy of Sciences, 1 Beichen West Road, Chaoyang District, Beijing, 100101 China; Beijing Institutes of Life Science, Chinese Academy of Sciences, Beijing, 100101 China

**Keywords:** Chemoreceptor, Orthoptera, Hemimetabolous, Peripheral olfactory system, Aggregative behavior, Phase polyphenism

## Abstract

Locusts represent the excellent model of insect olfaction because the animals are equipped with an unusual olfactory system and display remarkable density-dependent olfactory plasticity. However, information regarding receptor molecules involved in the olfactory perception of locusts is very limited. On the basis of genome sequence and antennal transcriptome of the migratory locust, we conduct the identification and functional analysis of two olfactory receptor families: odorant receptors (ORs) and ionotropic receptors (IRs). In the migratory locust, there is an expansion of OR family (142 ORs) while distinctly lower number of IR genes (32 IRs) compared to the repertoires of other insects. The number of the locust OR genes is much less than that of glomeruli in antennal lobe, challenging the general principle of the “one glomerulus-one receptor” observed in other insects. Most OR genes are found in tandem arrays, forming two large lineage-specific subfamilies in the phylogenetic tree. The “divergent IR” subfamily displays a significant contraction, and most of the IRs belong to the “antennal IR” subfamily in the locust. Most ORs/IRs have olfactory-specific expression while some broadly- or internal-expressed members are also found. Differing from holometabolous insects, the migratory locust contains very similar expression profiles of ORs/IRs between nymph and adult stages. RNA interference and behavioral assays indicate that an OR-based signaling pathway, not IR-based, mediates the attraction of locusts to aggregation pheromones. These discoveries provide insights into the unusual olfactory system of locusts and enhance our understanding of the evolution of insect olfaction.

**Electronic supplementary material:**

The online version of this article (doi:10.1007/s00018-015-2009-9) contains supplementary material, which is available to authorized users.

## Introduction

Olfaction plays a critical role in animal survival and reproduction, such as foraging, aggregation, predator avoidance, and mating [1, 2]. To adapt diverse environments and ecological niches, insects have evolved a wide variety of olfaction-based behaviors and diverse olfactory systems, which can be reflected by the morphological characteristics of antennae, olfactory sensilla type, olfactory receptor repertoire, and antennal lobe (AL) architecture [2–4]. Despite this diversity, a general principle has been proposed in the organization of insect olfactory pathway, from the activation of an olfactory sensory neuron (OSN) expressing one given OR, to the projection in one single glomerulus in AL, finally to the coding in higher brain centers. However, it remains unknown whether this general principle applies to all insect species because studies on important insect taxa, particularly insects undergoing incomplete metamorphosis, have not been conducted.

The identification of genes encoding odorant receptor (OR) and ionotropic receptor (IR) families is a key step toward understanding the characteristics and evolution of insect olfactory systems. Insects mainly rely on OR and IR genes to perform the long-range detection of volatile molecules [5, 6]. Insect ORs are ligand-gated ion channels and novel seven transmembrane domain proteins with an inverted topology compared with mammalian ORs [7–9]. These receptors are heteromultimers composed of at least one odorant-specific, highly divergent OR subunit (ORx) and a ubiquitous coreceptor *Orco* [10]. *Orco* is highly conserved across insect species and is essential for trafficking, localization and functioning of co-expressed ORx [9, 11]. OR repertoires have been identified in Diptera, Hymenoptera, Lepidoptera, Coleoptera, Hemiptera and Blattodea through whole-genome sequencing. The number of OR genes varies considerably from 10 in *Pediculus humanus humanus* [12] to 170 in *Apis mellifera* [13] and up to 352 in *Camponotus floridanus* [14], reflecting extensive gene gain and loss over the evolution of insect ORs. The origin of the OR family might be the adaptation of flying insects to the rapid spread and diversification of vegetation, with *Orco* being present before the appearance of ORs [5]. IRs, a highly divergent family of ionotropic glutamate receptors (iGluRs), represent a second family of insect chemoreceptors [15]. These receptors are also ligand-gated ion channels with three transmembrane domains. IR genes are found in olfactory organs across Protostomia; therefore, IRs likely represent an ancestral protostome chemosensory receptor family [16]. IRs can be further classified into two sub-families: conserved “antennal IRs” involved in olfaction and species-specific “divergent IRs”, which are detected in *Drosophila* gustatory organs rather than olfactory organs and function as candidate gustatory and pheromone receptors [16, 17]. The coreceptors of IRs, *IR8a* and *IR25a*, which are broadly expressed and analogous to the *Orco*, play an essential role in tuning IRs sensory cilia targeting and IR-based sensory channels [18]. However, the related studies of ORs/IRs mainly focus on holometabolous insects, such as flies, mosquitoes, and moths. The olfactory systems of more insect species should be examined to enhance our understanding to the olfactory coding and evolution of insect olfaction.

Locusts, which are a representative species of hemimetabolous insects, have been regarded as excellent model for studying insect olfaction because of their unusual olfactory system and striking density-dependent olfactory plasticity [1, 19, 20]. The organization of AL, which is the first-order olfactory center of insect brain, drastically differs in locusts compared with other insect species [20, 21]. In most insects, the number of glomeruli ranges from 50 to 200 [1]. Insects share a characteristic related to OSNs expressing a specific OR; similar to projection neuron (PN), each OSN project to a glomerulus [1, 22]. However, ALs in locusts display microglomerular organization with thousands of microglomeruli in each AL; similar to PNs, OSNs are highly branched in ALs and target multiple microglomeruli [1, 21, 23]. Differing from other insect species, locusts can add more microglomeruli into ALs and add new OSNs each time these insects molt, but the number of PNs remains constant as locusts age [24, 25]. As such, the olfactory system of locusts has been called the “puzzle” of insect olfactory evolution [1, 20]. Locusts also exhibit a striking phase polyphenism consisting of “solitarious” phase and “gregarious” phases. Olfactory preferences of solitarious and gregarious locusts can rapidly change as phase transition occurs [19, 26]. This phase-related olfactory plasticity has been regarded as a critical trigger of large swarm formation.

Several peripheral olfactory genes, including *CSP*s and *LmigTo1*, and two neurotransmitters, namely, octopamine and tyramine, have been suggested to play important roles in phase-dependent olfactory plasticity of the migratory locust, *Locusta migratoria* [19, 26]. Olfactory plasticity of locusts should be understood in terms of olfactory receptors because chemoreceptor family is also involved in the regulation of insect olfactory plasticity [27, 28]. However, information on locust chemoreceptors is very limited; thus far, only seven ORs and two IRs have been identified in two locust species, *L. migratoria* and *Schistocerca gregaria* [29–31].

The complete whole-genome sequence of the migratory locust provides insights into molecular characteristics related to the olfactory system [32]. In this study, the data from locust genome and transcriptome were integrated to perform manual annotation and characterization of the OR and IR families. Tissue- and development-specific expression profiles of these genes were also investigated. The specific olfactory signaling pathway responsible for the attractive behavior of the locusts was also identified via a gene silence technique and a behavioral assay. This study provides basis to understand the mechanism of locust odor coding and the evolution of insect olfaction.

## Materials and methods

### Locust rearing

The migratory locusts used in this research were obtained from colonies maintained in the Institute of Zoology, Chinese Academy of Sciences, Beijing, China. Gregarious nymphs were cultured in large boxes (40 cm × 40 cm × 40 cm) at a density of 500–1000 insects per cage. These colonies were subjected to a 14 h light/10 h dark cycle at 30 ± 2 °C and fed with fresh wheat seedlings and bran.

### RNA isolation

Total RNA was isolated using an RNAeasy mini kit (Qiagen, Hilden, Germany) according to the manufacturer’s instructions and treated with DNase I (Qiagen, Hilden, Germany) to digest the remaining genomic DNA. RNA concentration was determined using an ND-1000 spectrophotometer (Nanodrop, Wilmington, DE, USA). RNA integrity was confirmed through 1 % agarose gel electrophoresis.

### Sequencing, de novo assembly and analysis of antennal transcriptome

Antennal samples were hand-dissected from fourth-instar gregarious nymphs (at 3 days after molting; 20 individuals; male:female = 1:1) and immediately placed in liquid nitrogen. Total RNA was isolated using the method described above. mRNA was isolated and cDNA library was prepared using Illumina TruSeq RNA Sample Preparation Kits V2 (Illumina Inc., San Diego, CA, USA). The library was then deeply sequenced using Illumina HiSeq™ 2000.

After sequencing was performed, to de novo assemble the transcripts, raw reads were preprocessed by filtering low-quality reads and adaptor contamination by using Trimmomatic (version 0.30) with the parameters: “ILLUMI NACLIP:/path/to/adaptor/sequence.fa:2:8:6 SLIDIN GWINDOW:4:15 MINLE N:40”. The Trinity pipeline (version r2013-02-25) was used to assemble the filtered transcriptome data with default parameters [33]. To reduce redundancy, we firstly use TGICL (version 2.1) to cluster the assembly according to pairwise sequence similarity; the consensus sequences were produced for each cluster. The results were further filtered by cd-hit [34] (version v4.6.1-2012-08-27) with the parameter “-c 0.95”, which clustered the sequences based on the short word and selected one longest sequence as the representative for each cluster.

The raw RNAseq reads were mapped to the locust genome by Tophat (version 2.0.13) [35]. The number of reads that mapped to each gene model was counted by Htseq. The gene expression levels were measured by the RPKM (reads per kilobase per million), which was calculated by in-house PERL script. The transcript abundances of genes in the antennal RNAseq were compared with previously published whole body RNAseq data [36]. To minimize the influence of differences in the RNA output size between samples, the number of total reads was normalized by multiplication with normalization factors, as suggested by Robinson [37]. Differentially expressed genes were detected using the method described by Chen et al. [36], which was constructed based on the Poisson distribution and eliminated the influences of the RNA output size, sequencing depth, and gene length. Differentially expressed genes were determined by setting a fold-change cutoff of at least 2 and a false discovery rate (FDR) cutoff of 1E−5. Enrichment analysis for the supplied gene list was performed based on an algorithm presented by GOstat [38], with the whole annotated gene set as the background. The *p* value was approximated using the *χ*^2^ test. The Fisher’s exact test was used when any expected value of count was below 5. If one GO item was an ancestor of another item and the enriched gene list of these two items was the same, the ancestral item was deleted from the results. To adjust for multiple tests, we calculated the FDR via the Benjamini–Hochberg method for each class.

### *LmigOR/IR* gene identification

To comprehensively identify OR/IR genes, we adopted two strategies. First, we predicted the locust OR/IR genes from the recently sequenced locust genome using the protein profile implemented in AUGUSTUS. The identity of OR/IR amino acid sequence ranged from 15 to 99 % across insect species [13]; therefore, the traditional homolog searching method is not a straight-forward approach to search the genome. We collected the OR/IR protein sequences from four insect species: *Drosophila melanogaster*, *A. mellifera*, *Acyrthosiphon pisum* and *Tribolium castaneum*. For the identification of IRs, we also added the protein sequences from *Zootermopsis nevadensis*, which is closely related to locusts and displays an expansion of IR repertoire [39]. The multiple sequence alignment was performed with the Muscle program, whereas the gap-rich sequences were filtered by the prepareAlign program in AUGUSTUS. To get a good alignment, the protein sequences of IRs with length <500 amino acids were removed. Finally, the profile was generated using the msa2prfl.pl scripts; this profile was used to search the locust genome sequence with AUGUSTUS. The predicted OR/IR genes were further filtered when the best hit in NCBI/NR database was not an OR/IR gene. The predicted OR/IR genes were further manually curated utilizing the Appolo [40] and IGV [41]. Second, to predict more locust OR/IR genes, we also searched against the antennal transcriptome assembly via the protein profile method. The protein profile was constructed from all the locust OR/IR genes identified in the above-mentioned steps. The protein profile construction and search were performed with the HMMER version 3.1 program. The cutoff E value of the search was 0.01. The searched result was also filtered by the NCBI/NR database search as described in the abovementioned steps. Finally, the OR/IR sequences from both methods were merged to give a comprehensive locust OR/IR gene set.

### Phylogenetic analyses

Amino acid sequences of the selected ORs and iGluRs/IRs were aligned with the MAFFT (E-INS-I parameter) program. The alignments were then manually cleaned to obtain the final high-quality alignments. We used ProtTest to evaluate the optimal model of substitution to infer the phylogeny. Dendrograms were then calculated with the MrBayes v3.2.1 and RAxML programs before the trees were viewed and graphically edited with FigTree (http://tree.bio.ed.ac.uk/software/figtree). Bayesian analysis was performed via the WAG substitution model, four chains, two runs with 3 million generations. Trees were sampled each 100 generations. One-fourth of the 30,000 topologies were discarded as burn in, whereas the remaining topologies were used to calculate the posterior probabilities.

### Reverse transcription PCR (RT-PCR)

The antennae, maxillary palp, wing, leg, brain, testis, ovary, and fat body were dissected from fourth-instar gregarious nymphs aged 3–4 days after molting or from gregarious adults aged 7–8 days since eclosion. All samples were stored at −80 °C before RNA isolation. First-strand cDNAs were synthesized from 2 μg of total RNA with the oligo dT_(15)_ primer (Promega, Madison, USA) and the MMLV reverse transcriptase (Promega, Madison, USA). Subsequently, the cDNAs were used as templates for RT-PCR studies. The RT-PCR experiments were conducted in a thermal cycler (Eppendorf, Hamburg, Germany) and performed in a 40 μL reaction system, which contained 20 μL of r*Taq* mix (Takara, Dalian, China), 10 μmol of each primer, 2 μL of cDNA, and 16 μL of deionized water. The PCR parameters were 94 °C for 5 min, followed by 35 cycles of 94 °C for 30 s, the annealing temperature (primer-dependent) for 30 s, and 72 °C for 1 min, with final extension at 72 °C for 10 min. The RT-PCR products were analyzed on 1 % agarose gels and verified by DNA sequencing. To distinguish between genomic DNA and cDNA templates, primers were designed to span at least one intron. The ribosomal protein 49 (*rp49*) gene was provided as a control for the integrity of the cDNA templates. The primers were designed using the Primer 5.0 software and are listed in Table S1.

### RNA interference (RNAi)

Double-stranded RNAs (dsRNA) of green fluorescent protein (*GFP*), *LmigOrco*, *LmigIR8a* and *LmigIR25a* were synthesized with the T7 RiboMAX Express RNAi system (Promega, Madison, USA), following the manufacturer’s instructions. The concentration of dsRNA was determined with an ND-1000 spectrophotometer. The quality was verified by 1 % agarose gel electrophoresis. Fourth-instar gregarious nymphs aged 1 day after molting were separately injected with 9 μg of *dsGFP*, *dsLmigOrco*, or a mixture of *dsLmigIR8a* and *dsLmigIR25a* (*dsLmigIR8a*/*25a*) into the second ventral segment of the abdomen. Subsequently, the injected gregarious nymphs were marked and returned to the gregarious-rearing cages. After 3 days, the effects of RNAi were investigated by qRT-PCR, whereas the animal behavior was observed in a Y-tube olfactometer. The primers for dsRNA preparation were designed with the Primer 5.0 software and are listed in Table S2.

### Quantitative real-time PCR (qRT-PCR)

cDNA pools were generated from 2 μg of total RNA from each treatment; the pooled cDNA was used as the template for quantification. The qRT-PCR experiment was performed with a LightCycler^®^ 480 system (Roche, Mannheim, Germany). The reactions were performed in a mix containing 5 μL of SYBR Green I master (Roche, Mannheim, Germany), 5 μmol of each primer, 1 μL of the cDNA template, and 3 μL of deionized water. The thermal cycling was set for 1 cycle at 95 °C for 10 min, followed by 45 cycles at 95 °C for 10 s, 58 °C for 10 s and 72 °C for 20 s. The melting curve was analyzed to confirm the specificity of amplification. The relative expression level of the genes was normalized with the *rp49* gene. The expression data were analyzed with the equation: 2^−ΔΔC*t*^. The primers were designed using Primer 5.0 software and are shown in the Table S3.

### Behavioral assay

The experiment was performed in a glass Y-tube olfactometer at room temperature (25–30 °C). The system was equipped according to the instructions described in the literature [19]. Briefly, a constant airflow (300 mL/min) was filtered through activated charcoal and humidified with double-distilled deionized water, before it was introduced into each arm of the olfactometer. One lateral arm was connected to one empty bottle that served as the air control, whereas the other arm connected with one bottle containing the fourth-instar gregarious nymphs (40 individuals) as a volatile emission source. After observing 10 individuals, the two arms were exchanged to avoid unidirectionality. The initial choice for volatile or air was recorded when the locust walked for more than 5 cm into one of the arms. If locust did not make a choice within 3 min of being released, the outcome was recorded as “no choice”.

### Statistical analysis

Data from qRT-PCR were analyzed by Student’s *t* test. The choice of locusts in Y-tube olfactometer was analyzed by the Mann–Whitney *U* test. Differences were considered significant at *p* < 0.05. Data were analyzed using SPSS 16.0 (SPSS Inc., Chicago, IL, USA).

## Results

### Antennal transcriptome

To identify candidate OR/IR genes and determine their transcript abundance, we performed antennal transcriptome of fourth-instar gregarious nymphs via a deep-sequencing method and combining previous transcriptome data of adult antennae [32]. A total of 95,674,882 reads were generated: 81,356,958 (85 %) of which can be mapped to the locust genome (Table S4). A combined assembly pipeline included Trinity, TGICL and cd-hit-est; this pipeline was used to produce 84,160 contigs (Table S4). A total of 1281 genes were assigned for molecular function, 4159 for biological process, and 1834 for cellular component. The term “nucleotide binding” was the most represented (54 %) in the molecular function category, but the terms related to olfactory pathway were not enriched (Fig. S1).

To characterize further the antennal transcriptome, we compared the antennal transcriptome with the whole body transcriptome of fourth-instar nymphs [36]. The results showed that olfaction- and ribosome-related GO terms, such as “odorant binding”, “olfactory receptor activity”, and “structural constituent of ribosome”, were the most abundant upregulated genes of the antennal transcriptome (Table 1). Several GO categories, such as “oxidoreductase” and “catalytic activity”, were enriched in downregulated genes of the antennal transcriptome compared with the whole body transcriptome (Table 1).

**Table 1:** GO enrichment of differentially expressed genes between fourth-instar nymph antennae and whole-body transcriptomes

	GO ID	GO name	*p* value	GO classification
Up	GO:0005549	Odorant binding	2.37E−15	MF
	GO:0004984	Olfactory receptor activity	8.17E−13	MF
	GO:0007608	Sensory perception of smell	8.17E−13	BP
	GO:0004871	Signal transducer activity	1.45E−08	MF
	GO:0004888	Transmembrane signaling receptor activity	2.02E−08	MF
	GO:0038023	Signaling receptor activity	1.04E−07	MF
	GO:0004872	Receptor activity	1.09E−07	MF
	GO:0003735	Structural constituent of ribosome	3.81E−07	MF
	GO:0005840	Ribosome	5.27E−07	CC
	GO:0030529	Ribonucleoprotein complex	7.27E−06	CC
Down	GO:0055114	Oxidation–reduction process	7.06E−22	BP
	GO:0016491	Oxidoreductase activity	2.46E−18	MF
	GO:0003824	Catalytic activity	5.05E−12	MF
	GO:0008152	Metabolic process	1.69E−11	BP
	GO:0042302	Structural constituent of cuticle	2.26E−10	MF
	GO:0005344	Oxygen transporter activity	1.94E−09	MF
	GO:0022607	Cellular component assembly	9.50E−09	BP
	GO:0044282	Small molecule catabolic process	1.15E−08	BP
	GO:0071844	Cellular component assembly at cellular level	1.31E−08	BP
	GO:0065003	Macromolecular complex assembly	1.81E−08	BP

This table lists the top ten gene categories from the comparison of the expression levels of genes in the antennae RNAseq with those in the whole body RNAseq

*Up* GO terms enhanced in antennae, *Down* GO terms enhanced in whole bodies, *MF* molecular function, *BP* biological process, *CC* cellular component

### Identification and phylogenetic analysis of candidate odorant receptors

We identified a total of 142 candidate odorant receptors in the locust genome and antennal transcriptome, including 7 previously annotated ORs [29, 31], and we renamed these ORs in the present study. Newly identified *LmigORs* were consecutively named with Arabic numerals according to the scaffold locations and phylogenetic analysis of these genes. 54 % of these candidates (77 ORs) contained full-length open reading frames (ORF) with 6–7 transmembrane domains. Among 142 ORs, we only found a single pseudogene (*LmigOr100*) with a premature stop codon within an exon. Among the 142 candidate ORs, we detected the expression of 134 ORs in the antennal RNAseq except for *LmigOr5*, -*6*, -*25*, -*38*, -*83*, -*91*, -*115*, and -*141*. As expected, the *LmigOrco* gene exhibited the highest abundance (31.38 RPKM). The exon number, length, genomic location, and GenBank accession numbers of all 142 ORs can be seen in Table S5.

**Fig. 1:**
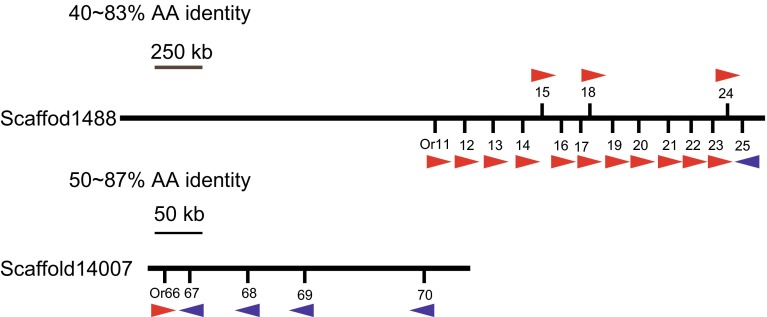
Genomic locations of partial *LmigOR* genes. The *central lines* represent two of scaffolds assembled in the locust genome. The orientation of gene transcription is shown with an *arrow*. The scaffold length (kb), gene locations and orientations are based on data from Release 2.4 of locust genome. *AA* amino acid

Gene mapping showed that most *LmigORs* were encoded by clusters of tandemly arrayed genes (Table S5). For example, 15 and 5 ORs were separately located on scaffolds 1488 and 14007 in two of the known perfect tandem gene arrays (Fig. 1). Some of these ORs had a high amino acid identity (up to 87 %), which reflected recent duplication events.

**Fig. 2:**
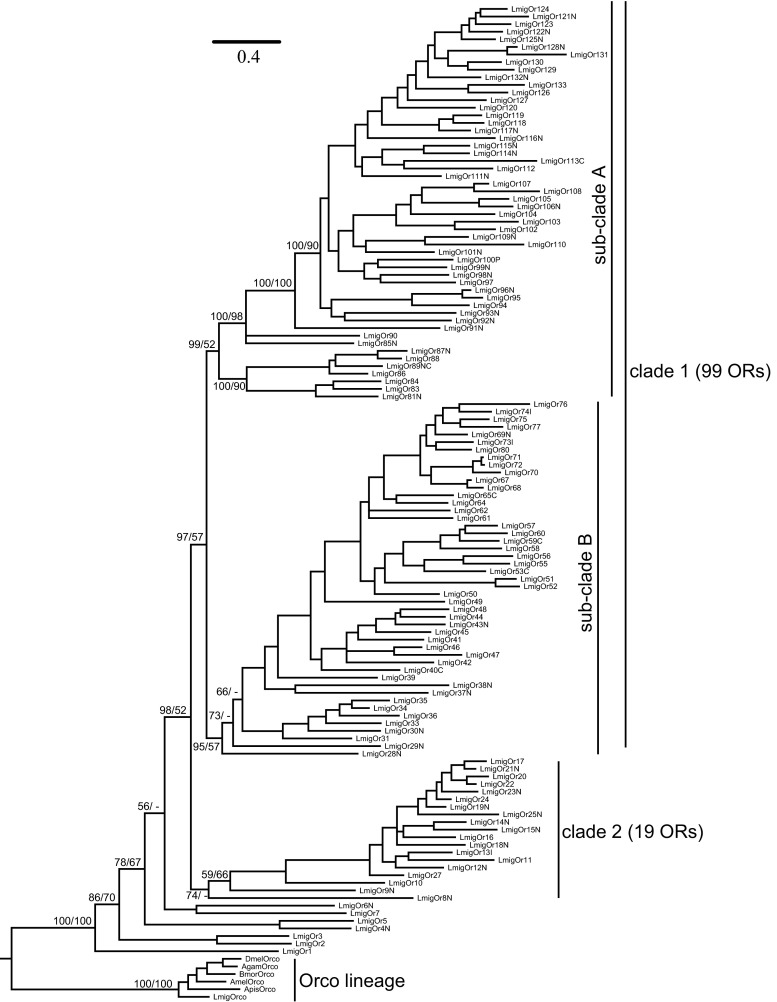
Phylogenetic analysis of *LmigORs*. 126 *LmigORs* (AA length > 250) were selected to build the tree. The dendrogram was generated by Bayesian analysis (WAG substitution model) and RAxML (JTT substitution model). Only support values for major branches and above 50 % are shown. The value before the *solidus* is given by Bayesian analysis whereas that after the *solidus* is given by RAxML method. Suffixes after OR names: *P* pseudogene, *N* N-terminal missing, *C* C-terminal missing, *I* internal region missing. Species abbreviations: Amel, *Apis mellifera*; Apis, *Acyrthosiphon pisum*; Bmor, *Bombyx mori*; Dmel, *Drosophila melanogaster*; Agam, *Anopheles gambiae*; Lmig, *Locusta migratoria*. The *scale bar* represents the expected changes per site

The phylogenetic tree of *LmigORs* was constructed based on Bayesian analysis, with the *Orco* lineage as the outgroup (Fig. 2). Additional ORs from other insect species other than *Orco* were not included because ‘‘orthologous’’ relationships between the 141 conventional *LmigORs* and other insect ORs were not detected (Fig. S2). The phylogenetic tree of *LmigORs* formed two large lineage-specific clades, including clade 1 (99 ORs) and clade 2 (19 ORs; Fig. 2). In the clade 2, 15 members were distributed on the scaffold1488 (Fig. 1). Based on the encoded protein sequences, the evolutionary relationships of these 15 genes conformed well to their locations on the scaffold. The clade 1 actually consisted of two main sub-clades: sub-clade A including 52 ORs and sub-clade B including 47 ORs (Fig. 2).

### Identification and phylogenetic analysis of candidate iGluR/IR genes

We identified 16 candidate iGluRs and 32 IRs from the locust genome, including four pseudogenes (*LmigIR5*, -*16*, -*23*, and *LmigiGluR11*) with premature stop codons. Unlike the OR genes located on the genome as tandem arrays, the iGluR/IR genes were widely dispersed throughout the locust genome (Table S5). As expected, we identified the orthologous genes of *DrosophilaIR8a* and *IR25a* from the locust genome; these genes were named *LmigIR8a* and *LmigIR25a*, which shared 46 % and 55 % amino acid identity with *DmelIR8a* and *DmelIR25a*, respectively. In the antennal RNAseq, we detected the expression of all *LmigIRs* except *IR7*, -*11*, -*13*, -*14*, -*15*, and -*16*. Among these IRs, *IR8a* and *IR25a* had the highest transcript abundance (8.38 and 11.00 RPKM, respectively). The exon number, length, genomic location, and GenBank accession numbers of all 48 iGluR/IR genes can be seen in Table S5.

The alignment of the amino acid sequences of *LmigiGluR*/*IR* genes showed that one or several key amino acids (arginine, threonine, and aspartate/glutamate) were absent in the predicted glutamate binding domains of most *LmigIR* genes, which was similar to other IRs [5, 15]. However, these key amino acids were conserved among most *LmigiGluR* members (Fig. S3).

**Fig. 3:**
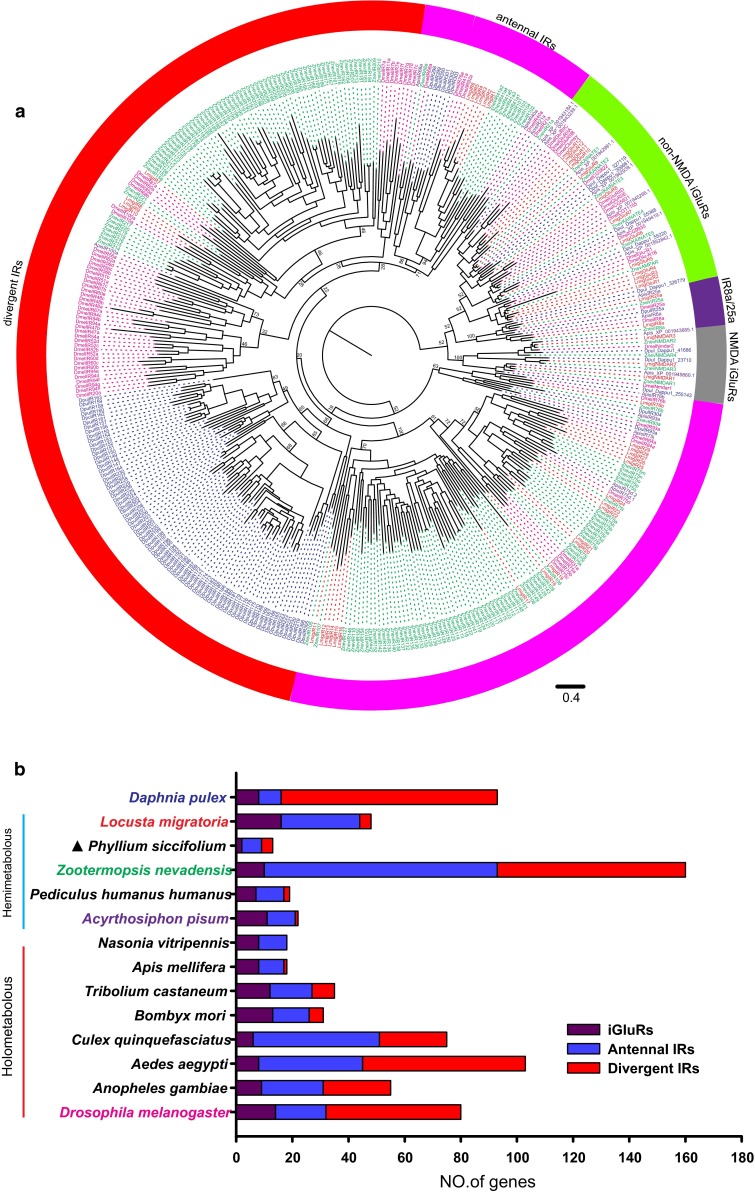
*iGluRs/IRs* identified in the locust. **a** Phylogenetic analysis of iGluRs/IRs. The dendrogram was generated using Bayesian analysis (WAG substitution model). Only support values for major branches are shown. Sequences of *Daphnia pulex, A. pisum,* and *D. melanogaster* are taken from reference 16; *Z. nevadensis* sequences are taken from reference 39. The *scale bar* represents the expected changes per site. **b** Histogram of the number of iGluR, antennal IR, and divergent IR genes identified in different species. The gene number of the different sub-families is counted according to reference 16 except for *P. siccifolium*, which is counted according to reference 5. The gene numbers in *Z. nevadensis* and *L. migratoria* are counted according to our in-house phylogenetic analysis. The species names involved in phylogenetic tree building are *colored*, and the *color* *pattern* is consistent with that in the tree. The organisms are sorted according to the evolutionary status. *Filled triangle,* genome is not sequenced

The phylogenetic tree of *LmigiGluR*/*IR* further supported our classification, in which 26 *LmigIRs* were well clustered into the conserved antennal IR sub-family (Fig. 3a). *LmigIR8a* and *LmigIR25a* were grouped close to a cluster of *IR8a/25a* genes from other insect species (Fig. 3a). Sixteen genes were classified into the iGluRs sub-family. There were only four *LmigIRs* belonging to the “divergent IR” subfamily (Fig. 3a). The number of divergent IR in the migratory locust was dramatically reduced compared to most other insect species (Fig. 3b).

### Tissue-specific expression of OR and iGluR/IR genes

To characterize further the candidate *LmigOR/IR* genes, we investigated their tissue-specific expression by RT-PCR analysis in several tissues of fourth-instar nymphs. Among 142 putative *LmigORs*, 108 OR genes were detected by RT-PCR in at least one tissue (antennae, maxillary palp, wing, and leg) of fourth-instar nymphs (Table 2; Fig. S4). More than half of these OR genes (63 of 108) were only expressed in olfactory organs (antennae and maxillary palp), including 52 antennae-specific ORs (Table 2; Fig. S4). Interestingly, 12 ORs showed a broad expression in all detected tissues (Table 2; Fig. S4).

**Table 2:** Summary of the tissue-specific expression of *LmigORs/IRs*

Pattern	*A*	*M*	*L*	*W*_OR_	*B*_IR_	No. of OR/IR	OR/IR (%)
1	●	○	○	○	○	52/4	48/19
2	●	●	○	○	○	11/1	10/5
3	●	○	○	●	●	16/3	15/14
4	●	○	●	○	○	3/1	3/5
5	●	●	○	●	●	10/3	9/14
6	●	●	●	○	○	1/2	1/9
7	●	○	●	●	●	3/0	3/0
8	●	●	●	●	●	12/4	11/19
9	○	●	●	●	●	0/1	0/5
10	○	●	○	○	○	0/1	0/5
11	○	●	○	○	●	0/1	0/5
						108/21	100/100

The expression of ORs and IRs was investigated in different tissues by RT-PCR

Wing tissue was investigated only for ORs, and brain tissue was investigated only for IRs. The tissue samples were dissected from fourth-instar gregarious nymphs aged 3–4 days after molting

*A* antennae, *M* maxillary palp, *L* leg, *W* wing, *B* brain, ● detectable, ○ undetectable

Among the 32 putative *LmigIRs*, 21 genes were detected by RT-PCR analysis in at least one tissue (antennae, maxillary palp, brain, and leg) of fourth-instar nymphs (Table 2; Fig. S5). Most of the putative *LmigIRs* (18/21) were detected in antennae tissue including 4 antennae-specific expressed IRs (Table 2; Fig. S5). Several *LmigIRs* were observed to have high expression in brain tissue (Fig. S5). A total of 14 iGluR genes were detectable and displayed a broad expression pattern; among these genes, 7 can be detected in antennae (Fig. S5). All the 3 NMDAR receptors showed brain-specific expression (Fig. S5).

**Fig. 4:**
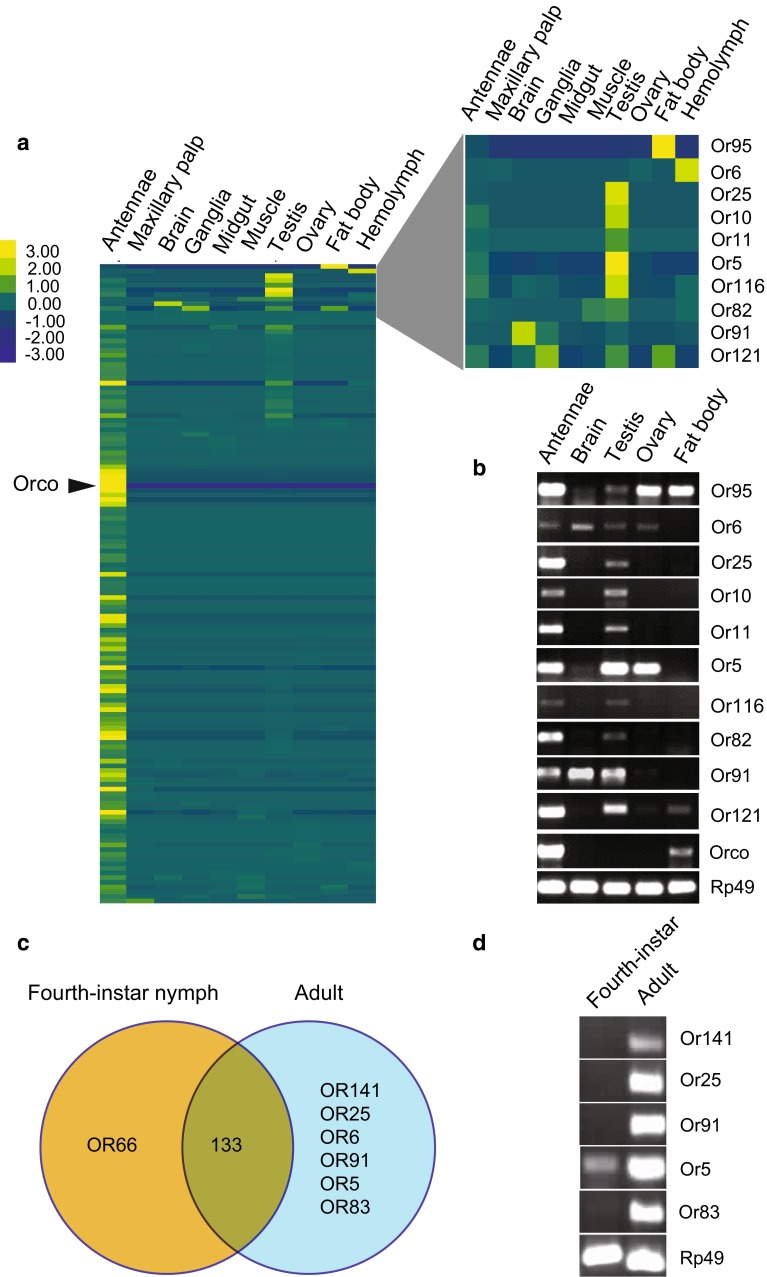
Expression of *LmigOR* genes in different adult tissues. **a** *Left* Heat map of *LmigOR* transcript abundances expressed in different adult tissues. *Right* Expanded view of the ORs expressed predominantly in internal tissues. **b** Expression of OR transcripts was confirmed by RT-PCR. The tissue samples were dissected from gregarious adults. **c** Comprehensive list of *LmigORs* expressed in adult and nymphal antennae RNAseq. 133, the number of ORs detected in both stages. **d** RT-PCR analysis of development-specific *LmigORs* in the adult and nymph antennae

Furthermore, the transcript abundances of candidate *LmigOR*/*IR* genes in various adult tissues were analyzed based on the transcriptomic data [32]. As expected, most of *LmigORs* had higher RPKM values in antennae than in other tissues (Fig. 4a). Interestingly, we found that 10 *LmigOR* genes were highly expressed in several internal tissues, especially in the testis (Fig. 4a). Additionally, the *LmigOR95* transcript abundance in the fat body (11.33 RPKM) was much higher than the mean expression level of antennae ORs (1.39 RPKM; Fig. 4a). In addition to antennae tissue, the *LmigOrco* transcript was also detected in the testis and ovary with very low transcript abundance (0.24 and 0.02 RPKM, respectively), but was absent in other investigated internal tissues (Fig. 4a). Subsequently, the expression of these 10 ORs and *Orco* was confirmed by RT-PCR (Fig. 4b). Moreover, RNAseq analysis showed that several *LmigIR* transcripts had a high expression level in the adult testis tissue other than the antennae (Fig. S6).

### Comparison of *LmigORs*/*IRs* expression in the antennae of nymphs and adults

We compared the expression patterns of *LmigOR/IR* genes between the fourth-instar nymphs and adults based on the antennal RNAseq data. The RNAseq analysis showed that 134 and 139 ORs were detected in the nymph and adult stages, respectively. Most of the *LmigORs* (133) were expressed in both stages (Fig. 4c). However, 6 *LmigORs* (*LmigOR5,* -*6,* -*25,* -*83,* -*91,* and -*141*) were adult specific, although only one OR, *LmigOR66*, displayed fourth-instar nymph-specific expression (Fig. 4c). The expression patterns of these development-specific *LmigORs* were confirmed by RT-PCR except for the expression of *LmigOR6* and -*66*, both of which had very low transcript abundance (0.09 and 0.18 RPKM, respectively; Fig. 4d). No development-specific *LmigIRs* were identified because all 26 IRs were detected in both stages (data not shown). In addition, we found 12 differentially expressed *LmigOR*/*IR* genes (fold-change > 2, FDR < 0.05) between the nymph and adult stages; 8 ORs and 2 IRs were upregulated in the nymphs, whereas 2 ORs and 1 IR were upregulated in the adults (Table S6).

### Olfactory signaling pathway linked with attractive behavior of gregarious locusts

**Fig. 5:**
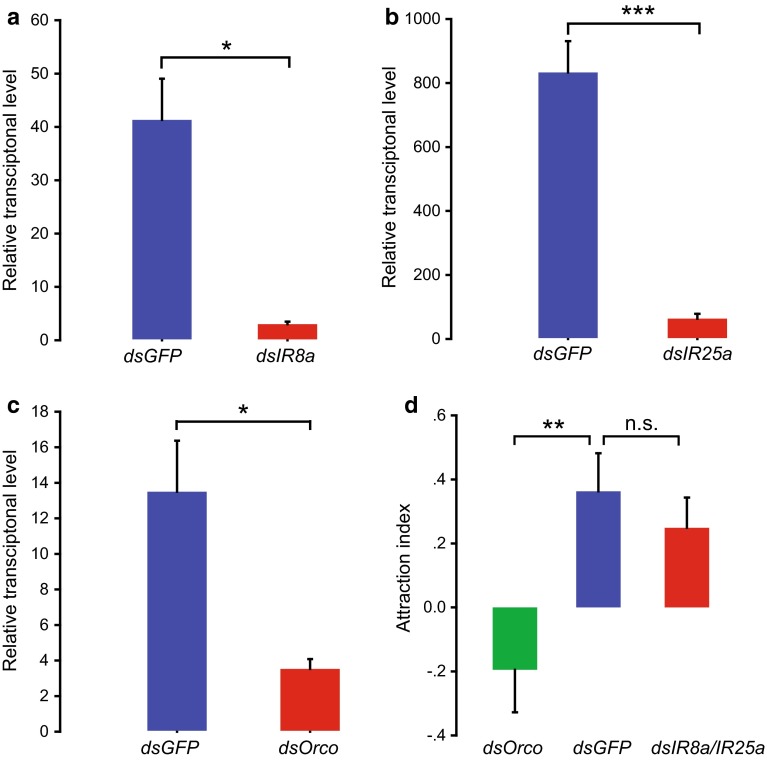
Behavioral changes of gregarious nymphs after RNAi. **a–c** Effects of RNAi of *LmigOrco, LmigIR8a*, and *LmigIR25a* genes (*n* = 4 or 6). Data conformed to a normal distribution as checked by the Shapiro–Wilk test, and statistical difference was evaluated by two-tailed Student’s *t-*test assuming unequal variance. **d** Dual-choice of gregarious nymphs in Y-tube olfactometer after injection of *dsGFP*, *dsLmigOrco*, or *dsLmigIR8a*/*LmigIR25a* (*n* = 57, 65, 56, respectively). Attraction index = (*N*_v_ − *N*_a_)/*N*_v_ + *N*_a_ + *N*_nc_; *N*_v_, the number of “choose volatiles”; *N*_a_, the number of “choose air”; *N*_nc_, the number of “no choice”. Statistical difference was evaluated by Mann–Whitney *U* test. *p < 0.05, **p < 0.01, ***p < 0.001, *n.s.*, not significant. Data are mean ± SEM

To distinguish the role of ORs from IRs in locust chemoreception, we tested behavioral responses of fourth-instar nymphs to aggregative pheromones from gregarious locusts via the RNA interferences of the coreceptor genes, *LmigOrco,* or *LmigIR8a*/*IR25a*. Compared with the controls, the relative mRNA expression levels of *LmigOrco*, *LmigIR8a*, and *LmigIR25a* significantly decreased by 74, 93 and 92 %, respectively (*t* = 3.635, 5.008, 8.637; *p* = 0.013, 0.015, 0.00013, respectively), after injecting their double-strand RNAs (Fig. 5a–c).

The Y-tube olfactometer assay was performed to determine the behavioral choice of fourth-instar gregarious nymphs to the odors of gregarious locusts. Compared with the *dsGFP* group, the attraction to the odors from the gregarious locusts was significantly decreased in the gregarious nymphs by ds*LmigOrco* injection (Mann–Whitney *U* = 666.5; *p* = 0.009) but remained unchanged in the locusts by ds*LmigIR8a/25a* injection (Mann–Whitney *U* = 806; *p* = 0.394; Fig. 5d).

## Discussion

The orthopterans are excellent models for investigating the evolution of insect olfaction because of their exclusive evolutionary trend of olfactory system [1, 20]. However, few investigations have focused on the components and functions of olfactory receptors in these species. Here, we identify a nearly complete set of OR/IR genes in the migratory locust. Their expression profiles and in vivo functions in olfactory behavior were also determined. Our discoveries provide the first extensive molecular insights into the unusual olfactory system of orthopteran insect.

### *LmigORs*/*IRs* repertoire in the locust

We performed a bioinformatic search for olfactory receptor genes based on the data of locust genome sequence combining with various transcriptomes. Transcriptome has the main advantage of facilitating novel gene discovery, especially for the species-specific chemoreceptor genes, whose detection is confounded in comparative genomic methods [42]. In addition, transcriptomic data help to modify the existing gene models. Finally, we identified a total of 142 candidate ORs and 32 IRs in the migratory locust. All these candidates are confirmed as true olfactory receptor genes by bioinformatics and tissue-specific expression analysis. Therefore, we believe that we have identified almost complete repertoire of *LmigOR/IR* genes.

Our results show an expansion of OR family (142 ORs) compared with the repertoires of other insects, except for species including honey bees (163 ORs) [13], beetles (341 ORs) [43], and ants (350 ORs) [14]. Most OR genes in the locust can be mapped onto genome in tandem arrays, which are also observed in other insect species [13, 44]. However, only a single pseudogene was found in the large *LmigORs* repertoire. The result reflects that the *LmigORs* repertoire may be subject to a low gene death rate, similar to that of transposons in the locust genome [32], according to the birth-and-death model of insect ORs [45]. Nevertheless, because the ORF of many ORs is incomplete, we cannot exclude the possibility that some of these ORs might be pseudogenes. This expansion of the *LimgOR* family has presumably provided the diversity of odorant receptors that allow locusts to recognize diverse odors. Indeed, locusts are polyphagous herbivores, which feed on a wide range of plants that emit complex and species-specific volatiles [46, 47]. In addition, locusts are surrounded by very complex odors emitted by other locust individuals and their feces when they form large swarms. Therefore, the expansion of the OR family in the locust may be adaptive for a voracious, generalist diet and complex olfactory cues.

Interestingly, the number of IR genes in the locust (32 IRs) is lower than that in other insect species, such as *A. gambiae* (46 IRs) [16], *D. melanogaster* (66 IRs) [16], and *Z. nevadensis* (150 IRs) [39]. The fruit fly has a similar number of OR and IR genes [16, 48]. However, the number of IR genes in the locust is much less than the number of ORs. Additionally, phylogenetic analysis shows that most *LmigIRs* (28/32) belong to the “antennal IRs” subfamily but only four “divergent IRs” were identified in the locust. Compared with other insect species, the number of divergent IRs is much lower in the migratory locust, implying that the contraction of “divergent IRs” subfamily in the locust may be the result of selection or drift during the evolution of the locust. A recent study on *Drosophila* suggested that divergent IRs are mainly expressed in peripheral and internal gustatory neurons, thereby implicating the involvement of this gene family in taste and food assessment [16]. Therefore, the contraction of “divergent IRs” subfamily might be related to an ecological adaptation of the gustatory sensory system in the locust. Future comparative and functional studies are needed to further explore the roles of *LmigIR*s in ecological adaption.

### Hypothesis of cluster organization in locust ALs

In the migratory locust, we identified a total of 142 ORs, which were much less than the number of glomeruli (~1000) within the AL [21]. Obviously, the locust olfactory system does not conform to the canonical relationship of one OR/one OSN/one glomerulus in other insect species studied so far [1, 22]. The organizational mode of one OR/one OSN/one glomerulus adopted by other insect species studied may ensure that odor representations are dispersed in the periphery but clustered centrally in the ALs [49].

To answer the question of how the locust species operates with the numerical disequilibrium between ORs and glomeruli in the olfactory system, we speculate that the locust AL may be divided into several anatomical or functional glomerular clusters (~142 clusters). These clusters have analogous roles to those of single glomeruli in other insect species. Each cluster is composed of a number of microglomeruli (~7 per cluster) that represent the same ORs, such that, all OSNs expressing the same ORs project their axons to the glomeruli of one cluster. The speculation of cluster organization in the locust ALs can be supported by evidence on the anatomical and physiological properties of the locust AL [20, 25]. Each glomerulus can be regarded as an individual dimension. Therefore, the huge addition of the temporal dimension in the locust AL can greatly expand the “coding space” of locusts [2]. However, if the cluster organization is true in the locust AL, the coding space in the locust would not be expanded as compared with those of other insects. Further experiments will be needed to verify this hypothesis.

### Tissue-specific expression of *LmigORs/IRs*

Our results showed that *LmigOR/IR* genes exhibit diverse expression patterns, which can be briefly classified into three types: olfactory-specific, internal-expressed, and broadly-expressed. Consistent with their roles in olfaction, most *LmigOR/IR* genes display olfactory-specific expression. However, we found that 11 conventional ORs are highly expressed in wings and legs other than in antennae. Given the co-expression of the *LmigOrco* gene in wings and legs, these ORs may be involved in olfaction, but unlike antennal ORs that respond to airborne volatiles, these ORs may perceive contacting pheromones [50]. Additionally, we found that several *LmigOR/IR* genes have higher expression in the testis rather than in antennae. These testis-enhanced *LmigORs/IRs* may be involved in endogenous ligand recognition, sperm chemotaxis, fertilization, or the activation of spermatozoa, as observed in mosquitoes [51]. Impressively, we observe the co-expression of a conventional OR (*LmigOR95*) and *LmigOrco* in the fat body. This phenomenon indicates that *LmigOR95* might play a role in monitoring internal metabolite levels by binding exogenous or endogenous ligands.

Furthermore, no phylogenetic pattern is associated with these internally or widely expressed OR genes in the phylogenetic tree, thereby suggesting that the ORs do not represent a single lineage of genes, but evolved independently. However, among the 142 putative *LmigORs*, 34 were not detected in all four tissues by RT-PCR, thereby indicating that these ORs may be not expressed in fourth-instar nymphs or are expressed at levels below the threshold of detection by RT-PCR. Instead, these ORs may manifest higher expression in other developmental stages such as the adult stage. In support of this hypothesis, *Or5,* -*82*, -*91,* and -*95* became detectable by RT-PCR in the adult antennae (Fig. 4b). Therefore, these *LmigORs* may have an important role in the adult stage, which is linked with the recognition of sexual pheromones and mating behaviors. Consequently, further research on these *LmigORs* is worthwhile to elucidate their roles in the non-olfactory tissues.

Consistent with the characterization of “antennal IRs”, most *LmigIRs* are highly expressed in the locust antennae. However, some *LmigIRs* are also detected in other tissues, such as the maxillary palp and brain, thereby indicating that *LmigIRs* may also have a gustatory function or may influence neuron activity in the CNS, as previously reported in *D. melanogaster* and *Spodoptera littoralis* [16, 52].

### Expression of *LmigORs/IRs* between nymphs and adults

Dynamic changes in the expression of olfactory receptors have been proposed to play important roles in olfactory plasticity depending on developmental stage, life experience and physiological state of insects [27, 53, 54]. The migratory locust, as a hemimetabolous insect species, has similar feeding habitats in nymphs and adults. Therefore, a similar expression repertoire for ORs (133/134) and IRs (26/26) is expected between the nymph and adult stages. By contrast, several related studies have shown large differences in the OR repertoire between the larval and adult stages in holometabolic species, such as *D. melanogaster* [55], *A. gambiae* [56], and *Bombyx mori* [57], all of which undergo a completely different lifestyle and feeding experiences from larva to adult. Additionally, six OR genes, including *LmigOR5,* -*6,* -*25,* -*83,* -*91,* and -*141*, are observed to be specifically expressed in the antennae of the adult locusts, thereby indicating potential important roles of these ORs in adult-specific behaviors, such as sexual communication or mating.

### OR-based signaling pathway mediates attractive behavior of gregarious locusts

The RNAi and behavioral assay indicated that OR-based signaling pathway, not IR-based, is mainly responsible for the attractive behavior of gregarious nymphs in the migratory locust. In *Drosophila*, ORs are usually located in the basiconic and trichoid sensilla, whereas IRs are expressed in the coeloconic sensilla [15]. IRs mainly respond to amines and acids that are largely ignored by ORs [58]. Therefore, ORs and IRs represent two evolutionarily and functionally distinct chemosensory subsystems [58]. Recent studies have indicated that *Orco* and *IR8a/25a* have a similar distribution pattern on the antennal sensilla of locust to that of *Drosophila*, that is, *Orco* is expressed in basiconic and trichoid sensilla, whereas *IR8a*/*IR25a* is located in coeloconic sensilla [30, 31]. The exclusive sensilla location of Orco and Irco in the locusts indicates that two different odor-sensing pathways may be present in the locust as in the fly. In the desert locust, aggregative pheromones are detected by basiconic sensilla that house OR-expressed OSNs [31, 59], being consistent with our results in the migratory locust. However, the identification of the conventional OR responsible for the detection of aggregative pheromones should be investigated in the future.

In brief, we characterized the unusual olfactory system of the migratory locust, including the large OR repertoire, the loss of “divergent IR”, and the possible existence of glomerular clusters in the AL. We also revealed expression patterns of broadly and internally expressed ORs/IRs, a similar expression repertoire of ORs/IRs between nymph and adult stages, and functional differentiation of ORs and IRs in the olfactory behavior of gregarious nymphs. Our study sheds new light on the understanding of unusual olfactory system of locusts and the evolution of insect olfaction.

## Electronic supplementary material

Supplementary material 1 (PDF 2489 kb)
