# Supplementary material for: Identification and functional analysis of olfactory receptor family reveal unusual characteristics of the olfactory system in the migratory locust
Source: Cell Mol Life Sci. 2015 Aug 12;72(22):4429–43. doi: 10.1007/s00018-015-2009-9 (PMC4611004; doi:10.1007/s00018-015-2009-9)
Supplement: Supplementary file 1 — Supplementary material 1 (PDF 2489 kb) [file 18_2015_2009_MOESM1_ESM.pdf]

Supplementary Information

Identification and functional analysis of olfactory receptor family reveal unusual characteristics of the olfactory system in the migratory locust

For: *Cellular and Molecular Life Sciences*

Zhifeng Wang[1#], Pengcheng Yang[2#], Dafeng Chen[1], Feng Jiang[2], Yan Li[2], Xianhui Wang[1*] and Le Kang[1, 2*]

[1]State Key Laboratory of Integrated Management of Pest Insects and Rodents, Institute of Zoology, Chinese Academy of Sciences, Beijing 100101, China; [2]Beijing Institutes of Life Science, Chinese Academy of Sciences, Beijing 100101, China.

[#] These authors contributed equally to this paper

*Correspondence to:

Dr. Le Kang

Institute of Zoology

Chinese Academy of Sciences

1 Beichen West Road, Chaoyang District

Beijing 100101, China

Tel: 86-10-64807219, Fax: 86-10-64807099

E-mail: lkang@ioz.ac.cn

OR

Dr. Xianhui Wang

Institute of Zoology

Chinese Academy of Sciences

1 Beichen West Road, Chaoyang District

Beijing 100101, China

Tel: 86-10-64807220, Fax: 86-10-64807099

E-mail: wangxh@ioz.ac.cn

Table S1 The primers for RT-PCR

**OR ID**	**Forward Primer（5' to 3'）**	**Reverse Primer（5' to 3'）**
LmigOR47	ATCCACCACTACACCCAC	GGAGCCGAAGAAGACG
LmigOR127	ATGGACGTGAGGAGGAAGAATT	ATCAGCATGAAGATGAGTAGGTTGT
LmigOR7	ACGACGGGACTGAAGGACAGC	GCCAGCATGATGAGCACGC
LmigOR80	CCATAGCCATGACGCAGTTCG	GGTAGAGGTGTCCAGCGGTGAG
LmigOR86	TGGCTGGGTGTTCAAGTACGG	GAGGCATTCATCAGCAGCAGG
LmigOR82	TGTTATTACGGCGGAGACG	GGGCGAAGTACGAGTAGGAG
LmigOR88	GACGCCTTCTTCGTGTCGC	CCCTGGAGATGGTGTAGAACTTG
LmigOR110	CACGCTCATTCTCAACTTCGC	TGCATGTGGGTCTTCCTCC
LmigOR63	CTGCCCTTCAACCACTTC	CGCTGTCCAGGAAAGAA
LmigOR141	GCTCTGCCTCATCTGGTT	TGGGATTCATCACGCTCT
LmigOR139	CAGGCACTTCTCAGGTC	TCCGTATGTTCCGTTT
LmigOR54	CGACGCAGTTCAAGATTC	CACGCAGGACGAGACA
LmigOR137	GGCTACGCCGTCTTCCA	GCCGTTCGCCCTCTTT
LmigOR79	AGTGTCCTCGTCGCTTGC	CCCTGTCGATGGGGTAGA
LmigOR136	ACTATGAGCCCCGTAGCGATG	CGAGAGGAAGGTCTCCCTGTTGA
LmigOR78	CTGCCTTCAGCAAGTAATG	GCGTTCACCAGCGAGA
LmigOR32	CCATGTGCATCGACCTCTT	GTGTTGTTCGCTGGTTGTAAT
LmigOR140	ATGCTGGCGTTCTTCCTC	CTCGCTGTCCTTCTCGTTG
LmigOR134	ACCTCGCCTCCGTCAA	GCCCGATACATCTCCACA
LmigOR26	ATTGTCAGGATTGCCGAAGAGG	CCAAACCAGCAGTAAATGAAGAGC
LmigOR117	CCCACCTACGAGCTGGTGTA	GAGCAGGCTGTGTCTGAACC
LmigOR25	ACCATCAGACCATCATTAGGAAC	GACATATTGACGACCGCCAC
LmigOR19	TGTCAGTCTCGCCAAAG	TGCCAGTGCCAGGTT
LmigOR122	TCCGAGCAGTGACAAG	CAAGACCTGGAGTAGCAT
LmigOR128	CCTCCGCCTCCTCATA	GAAACATTCTCGCTCAACA
LmigOR132	GAACTTATCGGCTATGGAA	CAGGACGCAGGGTATCA
LmigOR69	GCCGAGTATCCCGAGAAGAAG	TAGAGGTGTCCAGCGGTGAGCACCA
LmigOR115	TCTGGAGCAGAGAAGGTGGAG	ACATCGTAAGTATGTTGGTGAGGAG
LmigOR14	TTTCTGCGCCTTCCTCACC	AGCAAACGCCACCTTCTCG
LmigOR111	TGTTTTGGTAGATCGGAGGATG	GACGAAGGTCTCTTTGGAGAGTT
LmigOR30	ATCCTGACTCAAGTTTTGGGC	GGAGGCGTTGATCAGCTGT
LmigOR89	ATGGGCGTGTCGGTGTA	GATGTCCGTGAGGAACTGC
LmigOR28	TACCACCAGGAGTTGA	ATTTGCCTCAGAACAG
LmigOR101	GGTGTCCTTGTGGGTGC	CGAGCCGTAGGTGTTGTC
LmigOR9	ATGGGCTTCGTGGTGTGCG	TGTCTCCAAACCAGCAGTAGCAA
LmigOR59	CGTCGGCTTCCTGGTTT	GGACTTCCTCGCCTTCATT
LmigOR6	CAGAGCGACTTCAAACC	GCAAGGCTACACTCAAATAC
LmigOR125	ACAGTTCACAGTCCACATTAGGAG	GGCAGAGTGCATCAGCATC
LmigOR40	CCTCGTTCGTCTTCATCG	ACGGCAAGTGCTTCTCC

LmigOR91	TTCTTCGCCGTCACCAA	GGTTGAACAGTATTCCCACAA
LmigOR114	GGCTACGCCGTCTTCCA	TCGCCAGATTGTCGTTGAG
LmigOR93	ACTCCTCCTACCTCACCCTCAT	CAGTCGCTCGCTCTGTTCAAT
LmigOR43	CGCAGCTCGGTATTCTCA	CTGGCATTGGAAGGTACATGAAGCA
LmigOR15	AGGAAGCATATCGCTGAACTGG	GGAGAGAGGGAAGAAACCCG
LmigOR23	CGCTCGCCTACGCACTT	CGCAAGCATTACGACCAAG
LmigOR17	TGGGAGGACATCCCGGTGAT	CAGACTTTCGTGGCAATTTGAAACA
LmigOR37	ACAGCCTCCATCACTAA	AACGCATTCCGACTT
LmigOR10	GCCGCTTTCGCCTTCCA	CGTCGCCAAACCAGCAGTAG
LmigOR21	GGTGCTGGACTCCTTCAA	CACGTTCTCGCTTTCTGTT
LmigOR116	GGCTCGGGAACATAACCT	TCCTCAACAGTGCCAAATA
LmigOR76	CTGGGACAACAACCGGC	ATGACGCATTCACCAACGA
LmigOR20	GCCTCATCTCGCAGTTGC	CACATTGACGACCGACACA
LmigOR67	CAGAGCATGGTCTGGTACTTCGT	AGGCCTCCCTGTTGATAGGG
LmigOR27	GCAACTGCGACAGCAGCTCA	GACACGTTCAAAACCTGGACAAAG
LmigOR57	ACGTGTCGGTCGTGATAGTGGG	CTGGTGATTTCTTGGTGAGTGTGT
LmigOR74	CTCAGATGAAGGTCCTGGCGTC	CCTGTTGATGGGGTAGATGCG
LmigOR81	ATCAACTACATCTCCGCCCA	GCTCTCCAAGTAAACTTCACCTC
LmigOR12	GTGCTGTGCATGGTGTGGA	TCTCGCTCTCTGTGATGAGGT
LmigOR22	AAGCTGTACATGTGGGGCGG	CAGAAGCAGTAGTGAGCGTTTGAT
LmigOR55	GGTCGGCTGAGCGTGTTCTT	TCGTGGCTGGCGTCTTTTG
LmigOR24	CGTCCCTGTATTCTGAACTCC	CGTCACCGCGTCATAAGC
LmigOR13	ATCAGGCTATTATTAGGAACACCG	CGAAGAATGGCGAAGAAGGA
LmigOR124	ATCTACGAGACGCCCCACT	TTCTGCAACAGCACACCG
LmigOR51	AGTTCTTCTTCCTGGCGGTC	TGGTGATGGGGTAGAGGTGTC
LmigOR11	TGCATGTGGTGGCTGCTCTACC	GCTCACTTTCAGGCTCTCCGTT
LmigOR121	CTGTTGCAGAAAGAGGGAAGTA	ACCTGAAGTAGCGTCTCCTTG
LmigOR29	CAAAAGCCGACAAGAGAAGA	GCACGTGACACCACAGAGA
LmigOR35	GAGACGGGTGACTCGGAAATAA	CCTCGGCGTACCAGCAATA
LmigOR118	CGCAATGATGAAATCCG	TCACAACTGTACGCCGAG
LmigOR71	GCCAACTGGAGGAAACC	TGAGCCGCTACGAGGA
LmigOR72	CTACAGCCTGATGGTGCG	CGAGTATCACTTCCTGGTTCA
LmigOR75	AAGCACCTGGAGAACACTATGAG	ACGAAGGCTTCCCTGTTGATA
LmigOR64	TGCCGTTGATCGCGTACTCT	CCCCTGCTCCATGATGTTGTG
LmigOR77	GGCATGCTGCTGCTCATGTTCT	TCCGCCTGCTCGGTTACGTTGT
LmigOR34	TCCAGGGCAGAACAAAGCA	GGAACAGAGTGACGCAAATAATAAC
LmigOR94	TCTACGCTTACCAGACGGCA	AATAGGCAGCTTTCAGCACCT
LmigOR5	GTGTGGAGTTCCGCTCA	GAAGACAAGTATTTTCTGGG
LmigOR62	TGCTGCCCTTCAACCA	GGCGTCCAAGAACGAGA
LmigOR52	CGCTCTCCGTGGAGTTCTTCTTC	GGTTGGTGTTCAGGTCCTGGTTG
LmigOR16	CGAGCGATTACGAAAGAACATA	ACGAAGGACTCCCTGGAGAG
LmigOR96	GCCATTTCCGAGACCA	TTTCATCCTCCGAACCTAT
LmigOR120	GCAAAGAGGCGGGAGAAA	GCCCAAAGACGCAGAACAT

LmigOR39	ACAGTGGAGATACGGAGAGG	CGTTGATCAGCGAAACAA
LmigOR103	CTCCCCAGCCTACGAGATAAC	ACCACCTGCCCGAAGAAAC
LmigOR87	TGGATGGCTTCCTGGTG	AACTTGCTGGCGGTGAG
LmigOR133	TGTACACGCTGCAGGTCCTC	CCCCACTCTTGTCGGTTATGAT
LmigOR3	GCACCATCGTTTGCCTCTTCT	TCTCCTCAGCTTCCATTAATCTGTCT
LmigOR84	CGCCTTCTACGTGACGTTCATC	AGCCGGCATCTCTTGTTTTTTC
LmigOR31	TGGGTGGAGCAGCATAAGG	TTTGGTACAGTGTCACGCAAAT
LmigOR46	AACCGCGCCTGGTACTCGTT	CCGCTGCCCTCTGGATTAAAC
LmigOR126	GACGCCTTCTTCATCCGCCT	CGAGCATCACAATTCACCCAAC
LmigOR49	GGCTGGTTTTCGTGTGGTT	GCAATGGTCTCTGGGCTCT
LmigOR85	CTACGTCAGCGTCATGGTCTA	TCGCTCTTCGTCGTTGTAAT
LmigOR97	TAACGGGCGTCCACA	GCAAGGCTACACTCAAATAC
LmigOR83	GCGTCTGCTACTGGCTCAT	TGTTCTCCGCCGTCGTG
LmigOR95	GATACCGCTGACGACTGCTC	TGGCGATGACTTGGAAACAG
LmigOR130	CCGAGTATCCGAAGC	CAGTACATGCCCGTCT
LmigOR123	TTGGAAGACACCAGGTTAGAGTT	GCCATGTTGACGAATAAGAGAA
LmigOR92	GTGCTGGACAGGGAGTTCG	CGTAGCGGAAGCGTGGA
LmigOR129	AAACATGTACCTCTCACCCAC	CACCACAGCACCAACGA
LmigOR112	CCGAAAGGCAGACTGAGAGTG	CAGGAAGGTGGACCTGGACA
LmigOR70	TGGTGTTGATATTCACTTCGACG	TGCAAAAGCACAATCCTTCG
LmigOR107	GCTGGTAGGCAACACGAC	GCTCAACAAATCCGAAGG
LmigOR108	CCGCAAAGAGACGACTGTATG	GAGAGTGTGACTATTTTTCCCACC
LmigOR2	GATGTTTCTGCTATGTGACTG	GCTATTTTTCCAACCGTG
LmigOR105	CATCTCTCTCTCCTTGTTTGTAGT	AAATGGGCGGTCTGGTT
LmigOR102	GTGACGGTGTTGCTGTGG	GATGTATGCTATAATGGCTTGA
LmigOR41	TACTCGGTCGCCTACTTCGTGC	GCCTGTTGACGGGGTAGAGC
LmigOR1	CTGAGCAGAAAAGAACAACA	GACGAGTACAACGAGCCA
LmigOR106	GCGATGAAAGACCACG	GGAATGCCCACCAATA
LmigOR44	TGCCACTCGGTTTTCTACTG	ACGAAGGTGTCGCGGTT
LmigOR104	TTCACCTGGACCGTAAGGG	CGAGAAAGCAGAACAGAGCATT
LmigOrco	ACGCCAACGAGACCATCACC	ATCAGCCAGCAGCAGAACATCA
LmigGluR7	TGGGCATCGGCATCCTGTTTA	CCGTGTACGACGAGATGATGATGAG
LmigGluR2	GACTTCAGCAAGCCGTTCA	ACGATGCTCACGCCCAC
LmigGluR8	TCGAAATTCACTTAGCACTG	CTTGGCAACAAACCGTAG
LmigGluR13	CGCCATTGACGAGAAGAC	TAAAGATACTCCAAGATACGCC
LmigGluR12	TTCCGATGACCATAACGC	CCTGGAGGAGACGACGAG
LmigGluR11	GGCTGCCTTCCTCACCAA	GATGCCGTAGCCCTTGGA
LmigGluR10	GCTGTATCAACGGATGTGG	AGCGCCGTCTGGTACTCTATA
LmigGluR1	TCGTCGCCGCAACTACAC	CTCGCCCACCATTCCATC
LmigGluR4	CCCGAAGAACGACTACATCA	TGAGTCTCGCTTTCACCCT
LmigGluR9	TGAAACTAAGGAATGGGATG	AATGAACCAATGGCGAAC
LmigGluR5	ATCGTCAAGGATGGCAAGT	TGTCGTAGGGCGAGAAGC
LmigIR17	ATCATTGGAGCATTTGTTCAG	TGGATAGACCCTCAGGTTTTG

LmigIR27	GCTACGCCCACGACCTTA	GCCTAGCCTTAGCACTTTCC
LmigIR20	GCAACTCCCTGCTCTTCATTCT	CTGGTCCCGTAACTGAGGTCAT
LmigIR26	AACTTCACATTCAGCGTCCA	GCAGTAAGCAGTGTTATTCTCCA
LmigIR25	GAATCTGTCACAAGTGTCTGGGTCG	GCAGGAACGTGAACAGCAGCAC
LmigIR11	GCGGCGTTATTAAACCAG	CCATCTTCAGTAGTCTCCACC
LmigIR25a	ATTTATGAAGTGGCAGACGG	CTGTAGGGACTCCAACGATC
LmigIR76b	AGCAGGATTCGGTCAGTT	CTCAGCGATACAGCGTTT
LmigIR21	AGGGACAAGTGGGGAGAA	ACGGCGATGTAGGGGTG
LmigIR3	GCTGTCCTGGCTGCTGAT	CCAATCGTGAAGTAACCTCCTG
LmigIR24	GGCAGACTAACGGAACATT	GAGTAGGAGGTGAACAAGAAGA
LmigIR5	CCAGATACGAACCTCCCATAGACAC	GCCAGCCTTTACGAAGCACA
LmigIR8a	ATCAGTCTAGGTACAGGGTATGGGA	CCAGGGCGAGTCCGAATAGAGT
LmigIR1	TCTCCTCAGTGCTCACAGTACCGAA	CGCTCCCAGTAGATGTCCTCCC
LmigNMDAR1	CGAACCCAATACGAACAGCC	TGAACTTTAATCTCCACGTCATCTT
LmigNMDAR2	AAGCCTCAGTATGGGTAAATCT	GTTTCCATTTCTGCTATTTCC
LmigNMDAR3	GGCCCAACAAGTTCCTCA	TCGGTGTATCCGCTATCAAG
LmigIR9	CTTCAAGTATGTGCGAGGTA	ATGGAGATTGTGCGAAAC
LmigIR6	ACTGGGTTCTTGGTTCGG	TGAGGGAGTTGAGGTAGGC
LmigIR23	CTGATGTTCCTCCTGGTGCTG	GCTCCCGGTAGAAGTTCTTGAT
LmigIR10	GACGCTCCTATGGTATGCA	TCGTATCGCGGATTGTTT
LmigIR18	CCCCACCTTCGCCTTCTT	CCAGGGTATTCCAAACTTCACC
LmigIR29	CTCTTCGTGCTCATCCTGGTG	GGCGAGGTCCTTCTTGTTGTT
LmigIR7	ACATACGGTCCAACTCCC	CCAAAGACCTCCCAAGAT
LmigRp49	CGTAAACCGAAGGGAATTGA	GAAGAAACTGCATGGGCAAT

Table S2 The primers for dsRNA preparation

**Gene ID**	**Forward Primer（5' to 3'）**	**Reverse Primer（5' to 3'）**
GFP	CACAAGTTCAGCGTGTCCG	GTTCACCTTGATGCCGTTC
Orco	AGTTGGACGGTGCCTCTGA	CGAAGTAGCCGAACTTGGTGA
IR8a	GGTGCGGGACGAGAAGA	TTGGCGGTGAAGGTGGC
IR25a	GGATCAATTACCTCCAT	CAGCAGCAGCCTCA

Table S3 The primers for qRT-PCR

**Gene ID**	**Forward Primer（5' to 3'）**	**Reverse Primer（5' to 3'）**
IR8a	CGCCTACTGGCTGTTCGT	GTAGTTGATGCGGCTCTGG
IR25a	GCTGTTTGGGACTACCCAGTGAG	CGTATATCAGTTGCATCGCCTA
Orco	ACGCCAACGAGACCATCACC	ATCAGCCAGCAGCAGAACATCA
Rp49	CGTAAACCGAAGGGAATTGA	GAAGAAACTGCATGGGCAAT

Table S4 Summary of the *L. migratoria* antennae transcriptome

Total number of reads	95,674,882
Total base pairs (bp)	52,703,182
Q20 percentage	97%
N50 (bp)	1,033
GC percentage	42%
Mapping percentage of reads	85%
Total number of genes	14,169
Number of contigs	84,160
Mean RPKM	92.26
Mean OR RPKM	2.22
Mean IR RPKM	1.65

Table S5 Details of olfactory receptor family in the migratory locust

GeneID	Scaffold[a]	Location[b]	Strand[c]	AAs[d]	Exons[e]	Description	GenBank accession number	GenBank accession number[f]
LmigOr2	238	501491-600467	-	448	6	complete CDS	KP843269	
LmigOr91	271	1478533-1506384	-	368	6	N-missing	KP843314	
LmigOr98	333	464785-582416	+	401	9	N-missing	KP843339	
LmigOr134	443	2047940-2078830	-	244	6	N-missing	KP843288	
LmigOr118	533	70935-127513	-	410	10	complete CDS	KP843356	
LmigOrco	538	44737-222732	-	481	8	complete CDS	KP843368	AEX28370.1
LmigOr116	686	741353-824852	+	394	7	N-missing	KP843330	
LmigOr92	982	3684875-3736594	+	439	7	N-missing	KP843261	
LmigOr45	1055	904090-937157	-	423	6	complete CDS	KP843238	
LmigOr41	1055	572591-607380	-	453	7	complete CDS	KP843272	
LmigOr44	1055	799195-821782	-	467	5	complete CDS	KP843275	
LmigOr43	1055	734247-780877	-	384	7	N-missing	KP843321	
LmigOr42	1055	653778-685213	-	388	5	complete CDS	KP843326	
LmigOr7	1387	69329-93133	+	412	5	complete CDS	KP843296	KC689310.1
LmigOr10	1486	163983-300781	+	389	6	complete CDS	KP843328	
LmigOr16	1488	2275954-2305257	+	418	5	complete CDS	KP843234	
LmigOr25	1488	3203131-3261778	-	255	10	N-missing	KP843292	

LmigOr18	1488	2438956-2483245	+	270	6	N-missing	KP843295	
LmigOr14	1488	2076482-2111277	+	288	5	N-missing	KP843302	
LmigOr15	1488	2171660-2212303	+	385	6	N-missing	KP843322	
LmigOr23	1488	3049938-3096755	+	386	6	N-missing	KP843323	
LmigOr17	1488	2367169-2391282	+	388	6	complete CDS	KP843324	
LmigOr21	1488	2801019-2845820	+	394	6	N-missing	KP843329	
LmigOr20	1488	2681503-2719845	+	398	6	complete CDS	KP843332	
LmigOr12	1488	1785538-1841538	+	402	8	N-missing	KP843342	
LmigOr22	1488	2923062-2968451	+	404	6	complete CDS	KP843343	
LmigOr24	1488	3130831-3180030	+	405	6	complete CDS	KP843345	
LmigOr13	1488	1921300-2007337	+	405	7	complete CDS,internal gap	KP843348	
LmigOr11	1488	1629145-1728002	+	408	7	complete CDS	KP843352	
LmigOr19	1488	2547710-2580121	+	264	8	N-missing	KP843293	
LmigOr78	1679	508495-549465	-	236	5	N-missing	KP843284	
LmigOr73	1679	165243-181137	+	324	4	internal missing	KP843308	
LmigOr74	1679	229434-256653	-	401	5	complete CDS,internal gap	KP843197	
LmigOr71	1679	47519-72757	-	410	5	complete CDS	KP843358	
LmigOr72	1679	88323-126636	-	410	7	complete CDS	KP843359	
LmigOr75	1679	281596-319499	-	410	5	complete CDS	KP843360	

LmigOr77	1679	422866-441953	-	412	6	complete CDS	KP843362	
LmigOr76	1679	370790-385421	+	396	7	complete CDS	KP843331	
LmigOr54	1876	2613032-2645167	+	218	3	N,C-missing	KP843280	
LmigOr56	1876	2796333-2818998	-	360	3	complete CDS	KP843313	
LmigOr55	1876	2671305-2740342	-	404	5	complete CDS	KP843344	
LmigOr96	1996	166851-224506	-	419	7	N-missing	KP843235	
LmigOr49	2074	457977-521081	+	429	6	complete CDS	KP843251	
LmigOr93	2160	901080-962405	+	375	7	N-missing	KP843319	
LmigOr113	2173	201025-238325	+	287	9	C-missing	KP843301	
LmigOr82	2429	156676-195205	-	168	5	N-missing	KP843336	
LmigOr81	2429	39627-85347	-	402	6	N-missing	KP843341	
LmigOr83	2922	350677-418141	+	433	7	complete CDS	KP843257	
LmigOr6	3000	336106-412313	+	338	6	N-missing	KP843312	
LmigOr53	3090	568091-649196	-	370	7	C-missing	KP843316	
LmigOr8	3330	445305-529584	+	441	7	N-missing	KP843263	
LmigOr9	3330	624007-670892	-	327	6	N-missing	KP843310	
LmigOr39	3820	22764-35327	+	419	5	complete CDS	KP843237	
LmigOr103	4385	1426573-1482918	+	424	8	complete CDS	KP843239	
LmigOr105	4385	1677530-1723517	+	449	5	complete CDS	KP843270	
LmigOr102	4385	1099230-1158602	+	450	7	complete CDS	KP843271	

LmigOr106	4385	1794212-1889859	+	465	7	N-missing	KP843274	
LmigOr104	4385	1535341-1616355	+	468	7	complete CDS	KP843277	
LmigOr101	4385	871818-955493	+	326	6	N-missing	KP843309	
LmigOr38	4419	756478-799089	+	379	7	N-missing	KP843320	
LmigOr37	4419	816150-860995	+	388	7	N-missing	KP843327	
LmigOr79	4575	19177-64296	+	232	7	N-missing	KP843282	
LmigOr80	4575	99696-132121	-	403	5	complete CDS	KP843307	KC689311.1
LmigOr99	4791	763562-836092	-	372	7	N-missing	KP843318	
LmigOr132	5160	166457-192310	-	278	5	N-missing	KP843298	
LmigOr131	5160	98525-159409	+	472	8	complete CDS	KP843279	
LmigOr58	5360	303641-337701	+	412	5	complete CDS	KP843325	KF601292.1
LmigOr57	5360	193335-273569	+	401	5	complete CDS	KP843340	
LmigOr111	5393	435624-473767	+	291	5	N-missing	KP843303	
LmigOr135	6023	219867-264703	+	246	6	N-missing	KP843290	
LmigOr136	6533	355150-394944	-	236	5	N-missing	KP843283	
LmigOr40	6715	340712-354172	-	352	4	C-missing	KP843196	
LmigOr31	6750	584780-615844	+	428	4	complete CDS	KP843247	
LmigOr33	6750	878041-928225	+	471	5	complete CDS	KP843278	
LmigOr32	6750	761334-817552	+	239	5	N-missing	KP843286	
LmigOr30	6750	395299-466190	+	301	5	N-missing	KP843304	

LmigOr34	6750	1023732-1044365	+	411	5	complete CDS	KP843363	
LmigOr3	6847	693832-763498	-	425	9	complete CDS	KP843242	
LmigOr48	7047	2531-9009	+	425	3	complete CDS	KP843245	
LmigOr137	7241	29861-53231	+	227	2	N,C-missing	KP843281	
LmigOr100	8188	52361-113331	+	338	5	pseudogene, internal stop codon	KP843369	
LmigOr138	8816	901853-980784	+	184	6	N-missing	KP843232	
LmigOr107	8825	85647-176982	+	445	8	complete CDS	KP843267	
LmigOr108	8825	232958-300253	+	445	7	complete CDS	KP843268	
LmigOr109	8825	358800-469565	+	400	7	N-missing	KP843338	
LmigOr36	9061	552351-596564	+	429	6	complete CDS	KP843255	
LmigOr35	9061	440864-497901	+	408	6	complete CDS	KP843355	
LmigOr46	9088	7518-79092	+	428	6	complete CDS	KP843249	
LmigOr29	9954	232108-285831	-	408	7	N-missing	KP843354	
LmigOr59	10257	201395-228187	-	331	3	C-missing	KP843311	
LmigOr63	11555	587874-618599	+	203	7	N-missing	KP843243	
LmigOr62	11555	513072-550455	+	414	4	complete CDS	KP843366	
LmigOr28	11850	37028-72444	+	307	5	N-missing	KP843306	
LmigOr120	12507	205187-236456	-	419	6	complete CDS	KP843236	
LmigOr1	12612	196946-257942	-	454	5	complete CDS	KP843273	
LmigOr110	12663	253750-289575	-	453	6	complete CDS	KP843357	

LmigOr130	13899	367422-427306	+	434	7	complete CDS	KP843259	
LmigOr129	13899	255620-286887	+	439	5	complete CDS	KP843262	
LmigOr128	13899	61261-83069	-	275	3	N-missing	KP843297	
LmigOr66	14007	10877-34317	+	214	8	N-missing	KP843265	
LmigOr70	14007	283396-315733	-	443	7	complete CDS	KP843266	
LmigOr69	14007	159400-201032	-	278	7	N-missing	KP843299	
LmigOr67	14007	45332-67303	-	397	6	complete CDS	KP843333	
LmigOr68	14007	102495-136708	-	397	5	complete CDS	KP843334	
LmigOr52	14992	82249-128485	-	418	5	complete CDS	KP843233	
LmigOr51	14992	32746-60956	+	407	5	complete CDS	KP843350	
LmigOr84	15161	125544-161238	-	425	5	complete CDS	KP843244	
LmigOr85	15161	316873-354884	-	429	6	N-missing	KP843252	
LmigOr139	15183	506919-559918	+	215	5	N-missing	KP843276	
LmigOr50	16000	134387-146457	+	415	3	complete CDS	KP843367	
LmigOr119	16866	8160-57070	+	405	9	complete CDS	KP843347	
LmigOr87	17701	283800-337779	+	425	8	N-missing	KP843240	
LmigOr89	17701	505351-530687	+	307	5	N,C-missing	KP843305	
LmigOr86	17701	206486-243636	+	408	5	complete CDS	KP843315	KF601291.1
LmigOr88	17701	410446-468391	+	425	7	complete CDS	KP843346	
LmigOr94	17794	21-33012	+	412	5	complete CDS	KP843364	

LmigOr140	19403	265463-362508	-	240	6	N-missing	KP843287	
LmigOr115	19962	15516-118722	-	285	8	N-missing	KP843300	
LmigOr60	25114	15229-60648	+	407	4	complete CDS	KP843351	
LmigOr26	27030	21332-36321	-	244	4	N-missing	KP843289	
LmigOr27	27030	133353-173363	+	398	6	complete CDS	KP843335	
LmigOr117	27979	15002-57257	-	255	5	N-missing	KP843291	
LmigOr97	33453	11120-69220	+	431	6	complete CDS	KP843256	
LmigOr64	36282	60720-102804	-	410	5	complete CDS	KP843361	
LmigOr126	40625	703840-787960	+	428	6	complete CDS	KP843250	
LmigOr123	40625	233465-295456	+	438	7	complete CDS	KP843260	
LmigOr127	40625	821927-861395	+	436	7	complete CDS	KP843285	JQ766966.1
LmigOr122	40625	173720-194898	+	265	5	N-missing	KP843294	
LmigOr125	40625	448556-464223	+	344	4	N-missing	KP843195	
LmigOr121	40625	297-80234	+	409	6	N-missing	KP843353	
LmigOr124	40625	343449-372163	+	406	8	complete CDS	KP843349	
LmigOr114	40899	37330-87246	+	372	7	N-missing	KP843317	
LmigOr95	41620	22093-89277	-	435	7	complete CDS	KP843258	
LmigOr4	44466	126052-195223	+	430	10	N-missing	KP843253	
LmigOr61	53044	18890-40678	+	428	4	complete CDS	KP843248	
LmigOr133	53350	26954-67979	-	424	5	complete CDS	KP843241	

LmigOr112	64845	48398-91175	-	442	8	complete CDS	KP843264	
LmigOr5	64869	26768 30420	-	412	2	complete CDS	KP843365	
LmigOr65	81047	33085-82489	+	399	4	C-missing	KP843337	
LmigOr90	86831	50226-110881	+	426	9	complete CDS	KP843246	
LmigOr141	221049	3-31329	+	209	3	N,C-missing	KP843254	
LmigOr47	345274	1511-3865	+	448	3	complete CDS	KP843231	JQ766965.1
LmigGluR7	227	556058-805264	+	643	10	N,C-missing	KP843227	
LmigGluR2	27081	115531-286013	-	819	16	N,C-missing	KP843212	
LmigGluR8	7287	391956-829831	-	908	17	complete CDS	KP843215	
LmigGluR6	38360	70783-286653	-	871	16	N,C-missing	KP843198	
LmigGluR13	54802	6555-21522	+	281	5	N-missing	KP843202	
LmigGluR12	15170	552633-652241	+	898	13	N-missing	KP843203	
LmigGluR3	29044	17139-206648	+	988	22	N-missing	KP843204	
LmigGluR11	64094	18376-52051	-	275	5	pseudogene, internal stop codon	KP843205	
LmigGluR10	5239	1066904-1292862	+	811	14	N-missing	KP843206	
LmigGluR1	62691	68701-253547	+	623	10	N,C-missing	KP843207	
LmigGluR4	41206	53331-154916	+	307	7	N,C-missing	KP843208	
LmigGluR9	5239	1587556-1887820	+	872	16	N-missing	KP843209	
LmigGluR5	95182	808-67979	+	527	10	N-missing	KT279125	
LmigNMDAR1	12229	1252205-1637374	+	949	17	complete CDS	KP843218	

LmigNMDAR2	1105	202714-431587	+	482	9	N,C-missing	KP843221	
LmigNMDAR3	1413	2279235-3089514	+	917	18	complete CDS	KP843222	
LmigIR27	5021	360509-429829	+	439	5	N-missing	KP843226	
LmigIR20	3683	773933-793769	+	452	5	N,C-missing	KP843230	
LmigIR2	12456	33413-94519	-	487	8	N,C-missing	KP843220	
LmigIR26	2329	1751890-1835518	+	424	7	N-missing	KP843223	
LmigIR25	13596	160937-245800	-	239	4	N,C-missing	KP843228	
LmigIR11	1562	253454-334757	+	349	5	N-missing	KP843225	
LmigIR28	22259	60035-93301	-	397	7	N,C-missing	KP843229	
LmigIR19	17157	69711-104051	-	456	9	N-missing	KP843200	
LmigIR25a	3614	49520-222244	-	985	18	complete CDS	KP843219	AFP33229.1
LmigIR4	56782	66048-100927	-	317	6	N,C-missing	KP843201	
LmigIR76b	27332	21767-66888	-	535	3	complete CDS	KP843210	
LmigIR21	9690	85415-148190	-	314	5	N,C-missing	KP843211	
LmigIR3	32804	62812-106132	+	462	7	N,C-missing	KP843213	
LmigIR24	242	2440666-2517347	+	678	6	complete CDS	KP843214	
LmigIR5	36485	968-49555	-	402	6	pseudogene, internal stop codon	KP843216	
LmigIR8a	11680	234702-426375	+	898	15	complete CDS	KP843224	
LmigIR1	3044	172328-228222	+	319	6	N,C-missing	KP843217	
LmigIR8	5490	746608-762257	-	675	4	N-missing	KT279120	

LmigIR10	1821	112282-120520	-	680	5	N-missing	KT279121	
LmigIR7	2981	375672-377699	+	675	2	internal missing	KT279122	
LmigIR16	4576	31758-138292	-	921	12	pseudogene, internal stop codon	KT279123	
LmigIR9	229	650477-738419	-	493	12	complete CDS	KT279124	
LmigIR14	1346	19186-94007	+	631	9	N-missing	KT279126	
LmigIR15	5941	111385-185222	-	863	14	Internal missing	KT279127	
LmigIR6	35369	817-77847	+	650	9	complete CDS	KT279128	
LmigIR13	34178	87597-167238	-	687	11	N-missing	KT279129	
LmigIR23	2771	913074-997137	+	701	11	pseudogene, internal stop codon	KT279130	
LmigIR18	4654	99-107968	-	542	9	N-missing	KT279131	
LmigIR29	1368	689310-740469	-	408	8	N,C-missing	KT279132	
LmigIR12	34178	186027-260463	-	540	8	N-missing	KT279133	
LmigIR22	2771	1450774-1491367	-	563	8	internal missing	KT279134	
LmigIR17	1803	18755-108363	+	438	8	C-missing	KP843199	

a the v2.4 genome assembly scaffold
b the nucleotide range from the first position of the start codon to the last position of the stop codon in the scaffold
c coding strand, + being forward and - meaning reverse
d length of encoded amino acid sequence
e number of exons in coding region
f previously registered genes

Table S6 Differentially expressed ORs/IRs between gregarious fourth-instar and adult locusts (fold change > 2, FDR < 0.05)

	Gene ID	Fourth-instar	Adult	log2(adult/ fourth-instar)	FDR
**Fourth-instar-biased**	*LmigOr15*	2.81	0.73	-1.95	6.75E-04
	LmigOr79	1.74	0.52	-1.74	1.86E-02
	LmigOr55	3.57	1.21	-1.56	1.48E-03
	LmigOr16	9.01	3.14	-1.52	3.30E-07
	LmigOr60	5.33	1.98	-1.43	1.74E-04
	LmigOr108	11.86	4.56	-1.38	4.43E-08
	LmigOr52	2.93	1.13	-1.38	8.03E-03
	LmigOr96	2.20	0.89	-1.31	3.34E-02
	LmigIR28	3.60	1.22	-1.56	1.48E-03
	LmigIR6	4.70	2.19	-1.10	1.44 E-02
**Adult-biased**	*LmigOr83*	0.00	1.25	10.29	5.35E-04
	LmigOr5	0.00	1.84	10.85	9.90E-06
	LmigIR26	2.97	10.75	1.86	6.07E-11

FDR, false discovery rate; Data are RPKM values

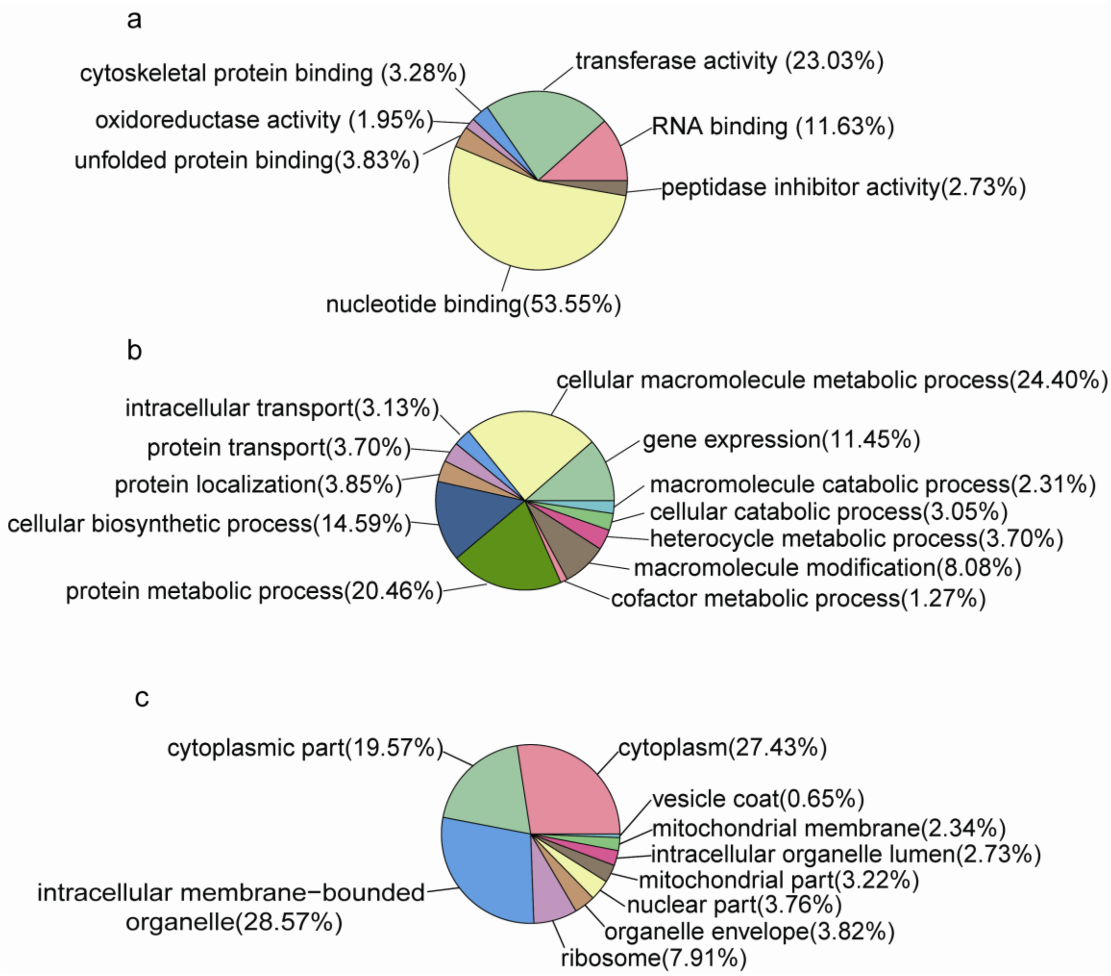

Figure S1 GO categories of genes expressed in antennal transcriptome. **a** molecular function (level 4 GO categorization). **b** biological process (level 4). **c** cellular component (level 4)

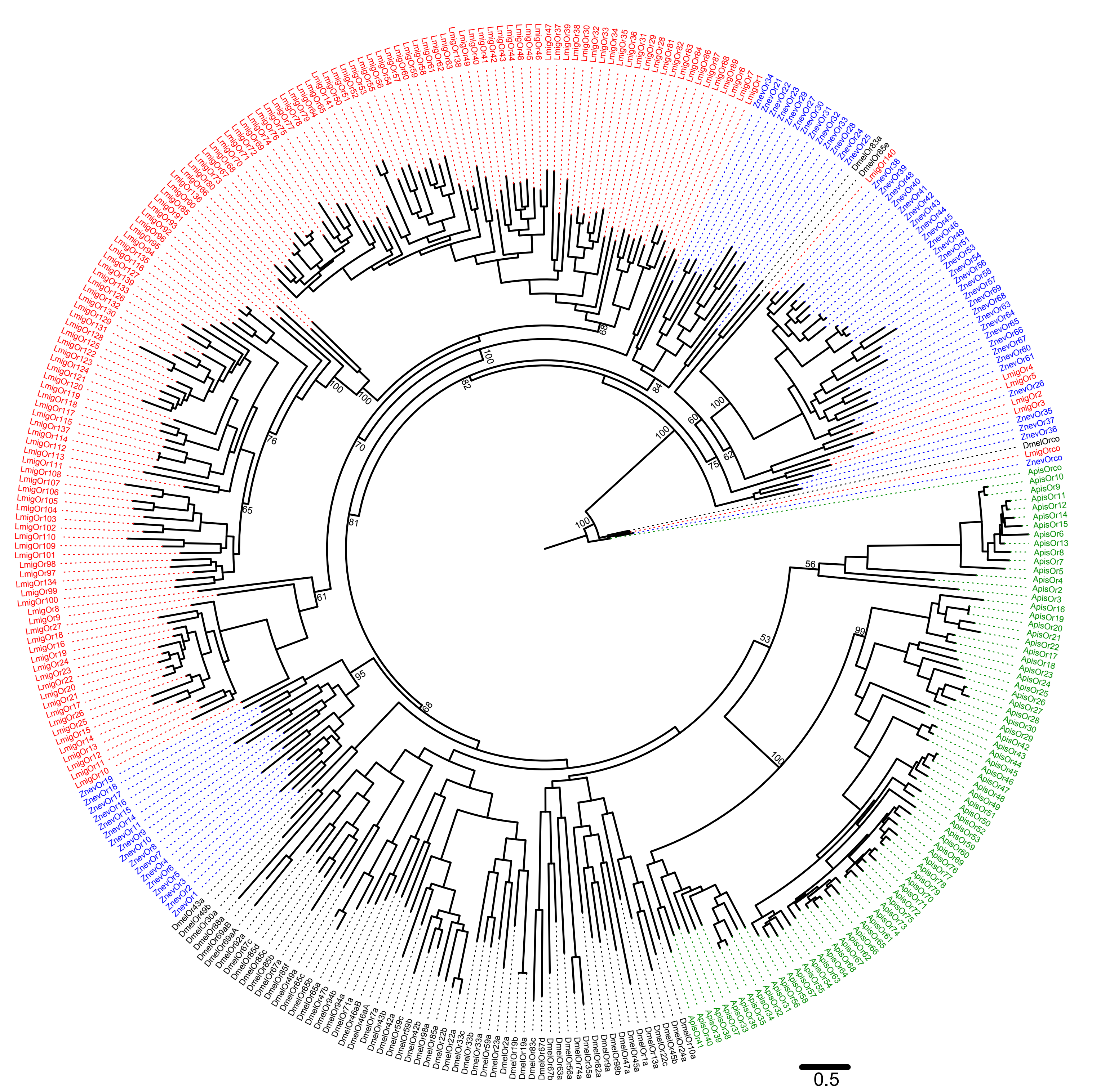

Figure S2 Species-specific expansions of OR repertoires. Phylogenetic analysis of all ORs from *D. melanogaster* (black), *A. pisum* (green), *Z. nevadensis* (blue) and *L. migratoria* (red). Sequences were aligned with MAFFT and the tree was built with MrBayes under the JTT model of substitution, with 6 million generations. Only support values for major branches are shown. The scale bar represents the expected changes per site

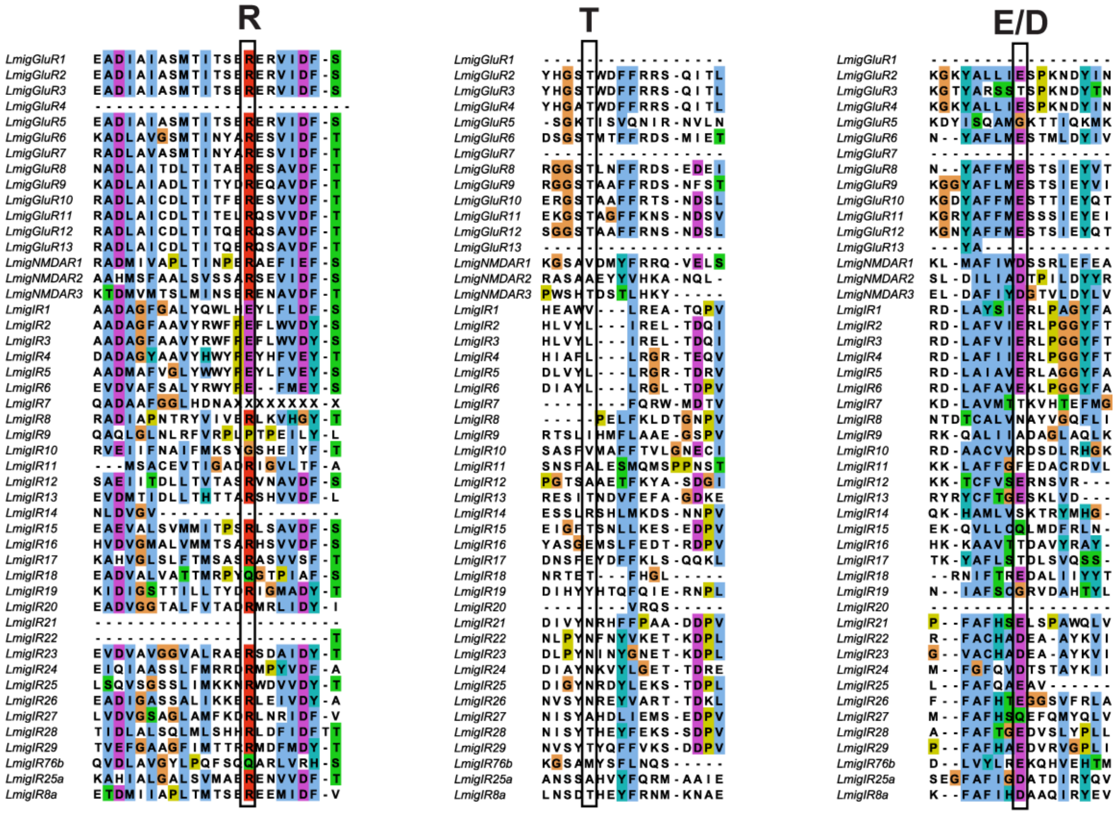

Figure S3 Excerpts of the amino acid sequences alignment among putative iGluR and IR genes. Three key glutamate-binding residues in iGluRs were boxed. Amino acid sequences were aligned using MAFFT program (E-INS-I parameter) and displayed and edited with Jalview 2.8.1. R, arginine; T, threonine; E/D, glutamate/aspartate

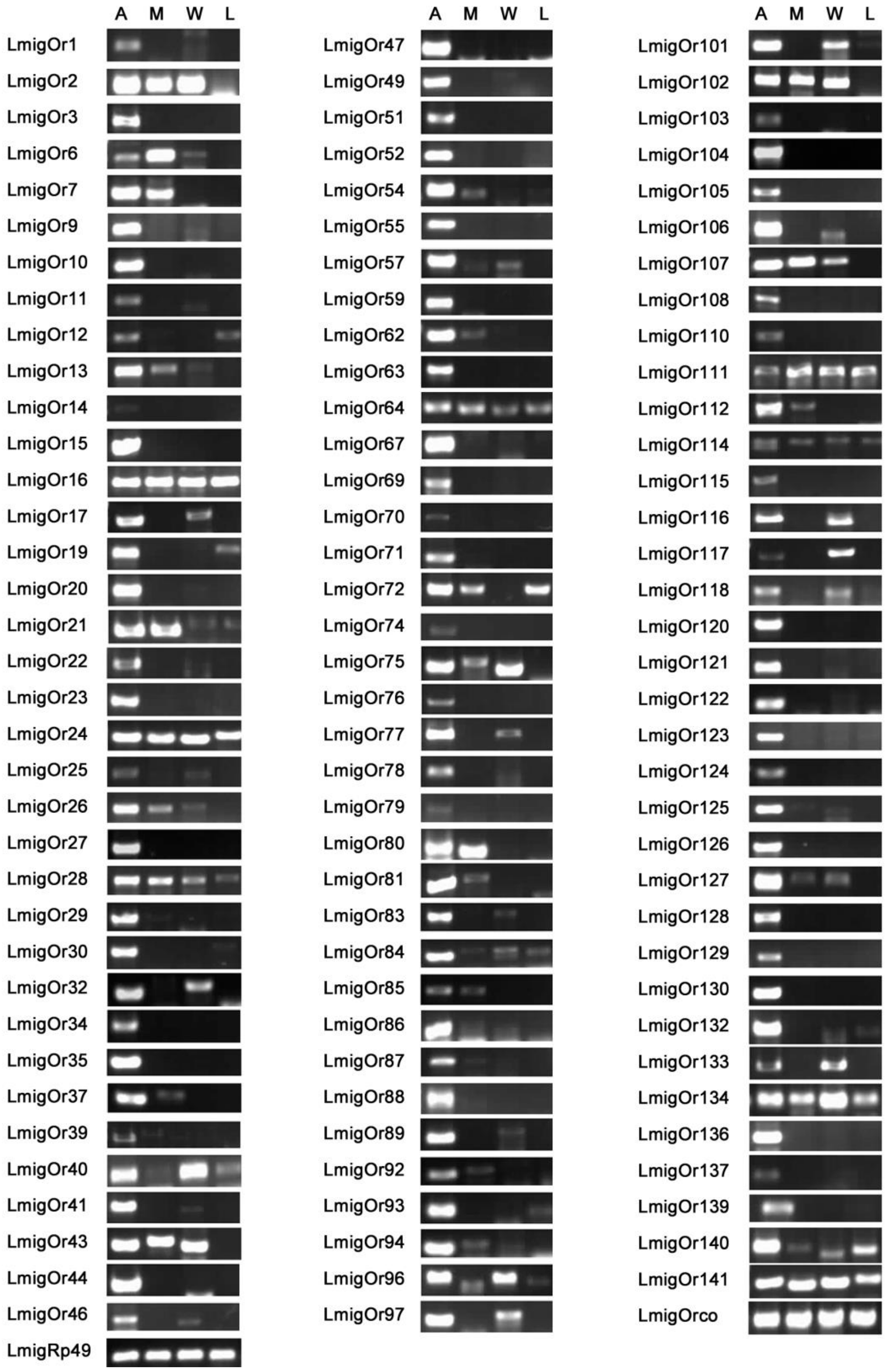

Figure S4 Tissue-specific expression of *LmigOR* genes. A, antennae; M, maxillary palp; W, wing; L, leg. *LmigRp49* gene is used as control for the integrity of the cDNA template. The tissue samples were dissected from fourth-instar gregarious nymphs

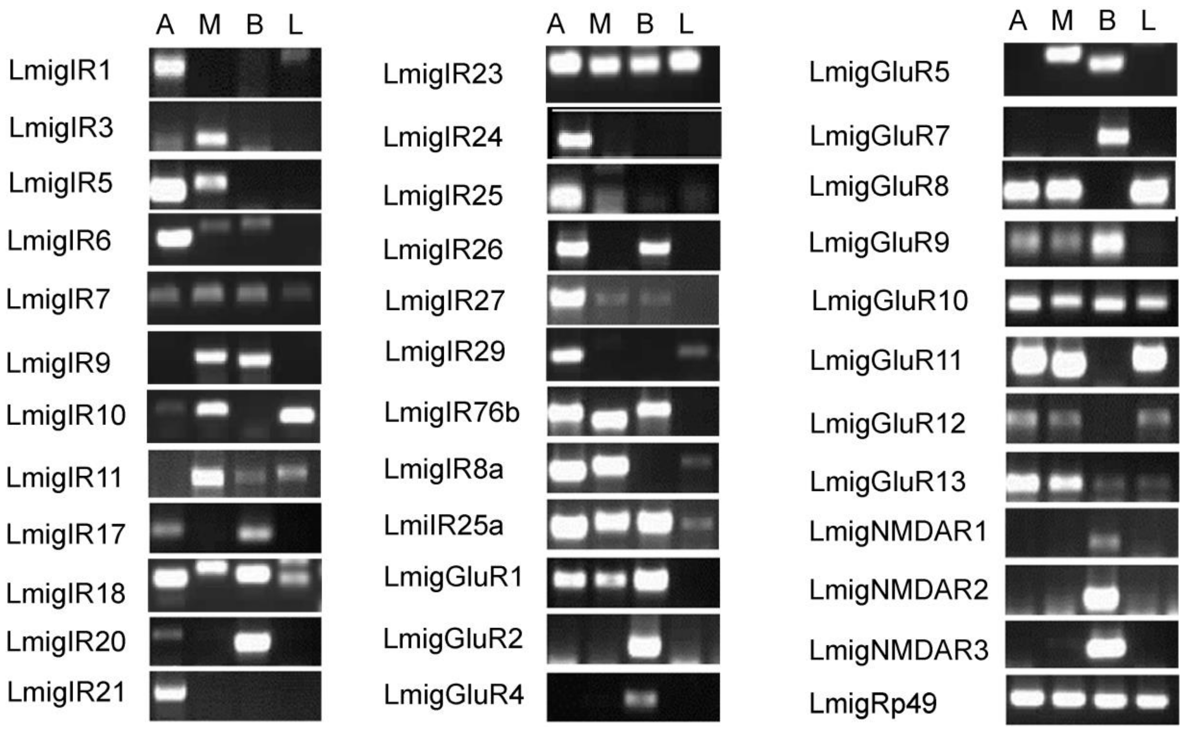

Figure S5 Tissue-specific expression of *LmigiGluR/IR* genes. A, antennae; M, maxillary palp; B, brain; L, leg. *LmigRp49* gene was used as control for the integrity of the cDNA template.The tissue samples were dissected from fourth-instar gregarious nymphs

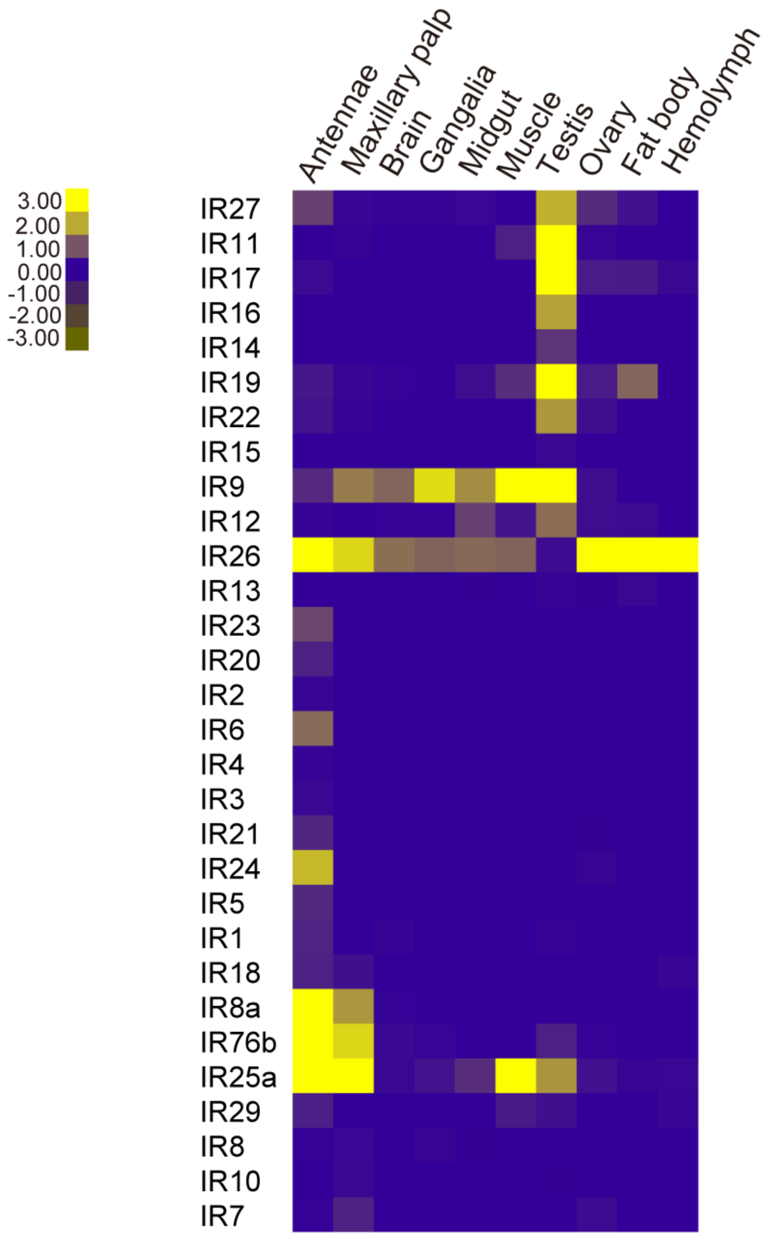

Figure S6 Heat map of *LmigIR* transcripts abundance in different adult tissues. The transcript abundances of *LmigIRs* were investigated based on RNAseq data of different tissues from gregarious adults aged 7–8 days since eclosion. Each row represents the RPKM values in different RNAseq data, and mean-centered by RPKM values
